# CAR-cell therapy in the era of solid tumor treatment: current challenges and emerging therapeutic advances

**DOI:** 10.1186/s12943-023-01723-z

**Published:** 2023-01-30

**Authors:** Karama Makni Maalej, Maysaloun Merhi, Varghese P. Inchakalody, Sarra Mestiri, Majid Alam, Cristina Maccalli, Honar Cherif, Shahab Uddin, Martin Steinhoff, Francesco M. Marincola, Said Dermime

**Affiliations:** 1 Translational Cancer Research Facility, National Center for Cancer Care and Research, Translational Research Institute, Hamad Medical Corporation, P.O. Box: 3050, Doha, Qatar; 2 Translational Research Institute, Academic Health System, Dermatology Institute, Hamad Medical Corporation, Doha, Qatar; 3 Department of Dermatology and Venereology, Hamad Medical Corporation, Doha, Qatar; 4 Laboratory of Immune and Biological Therapy, Research Department, Sidra Medicine, Doha, Qatar; 5 Department of Hematology, National Center for Cancer Care and Research, Hamad Medical Corporation, Doha, Qatar; 6 Department of Dermatology, Weill Cornell Medicine-Qatar, Doha, Qatar; 7 College of Medicine, Qatar University, Doha, Qatar; 8 Department of Dermatology, Weill Cornell Medicine, New York, USA; 9 Global Head of Research, Kite Pharma, Santa Monica, California USA; 10 College of Health and Life Sciences (CHLS), Hamad Bin Khalifa University, Doha, Qatar

**Keywords:** CAR-T, CAR-NK, CAR-M, Cellular immunotherapy, Solid tumors, Combined therapies

## Abstract

In the last decade, Chimeric Antigen Receptor (CAR)-T cell therapy has emerged as a promising immunotherapeutic approach to fight cancers. This approach consists of genetically engineered immune cells expressing a surface receptor, called CAR, that specifically targets antigens expressed on the surface of tumor cells. In hematological malignancies like leukemias, myeloma, and non-Hodgkin B-cell lymphomas, adoptive CAR-T cell therapy has shown efficacy in treating chemotherapy refractory patients. However, the value of this therapy remains inconclusive in the context of solid tumors and is restrained by several obstacles including limited tumor trafficking and infiltration, the presence of an immunosuppressive tumor microenvironment, as well as adverse events associated with such therapy. Recently, CAR-Natural Killer (CAR-NK) and CAR-macrophages (CAR-M) were introduced as a complement/alternative to CAR-T cell therapy for solid tumors. CAR-NK cells could be a favorable substitute for CAR-T cells since they do not require HLA compatibility and have limited toxicity. Additionally, CAR-NK cells might be generated in large scale from several sources which would suggest them as promising off-the-shelf product. CAR-M immunotherapy with its capabilities of phagocytosis, tumor-antigen presentation, and broad tumor infiltration, is currently being investigated. Here, we discuss the emerging role of CAR-T, CAR-NK, and CAR-M cells in solid tumors. We also highlight the advantages and drawbacks of CAR-NK and CAR-M cells compared to CAR-T cells. Finally, we suggest prospective solutions such as potential combination therapies to enhance the efficacy of CAR-cells immunotherapy.

## Introduction

Cancer presents a paramount health issue with increasing annual incidence and mortality rates [1]. Conventional therapeutic approaches involving surgery, radiation therapy and chemotherapy have major drawbacks and many patients with metastatic or recurrent disease still face dismal outcomes [2, 3]. In the last decade, various targeted treatments have considerably evolved owing to increasing knowledge in cancer molecular medicine and in immuno-oncology, allowing the development of precision medicine as a more specific and less toxic way to manage cancer [4]. Antitumor immunotherapy provided a major advance in the treatment of cancer by modulating the immune system to enhance its ability to recognize and destroy the malignant cells [5]. A broadly successful antitumor cellular immunotherapy approach consists of engineering immune cells to express cell surface receptor/s capable of recognizing antigens expressed on the surface of tumor cells and destroying them [6]. Subsequently, genetically modified immune cells are redirected through the Chimeric Antigen Receptor (CAR) to the tumor cells [7]. Currently, approved CAR-T cell therapy targets are mostly the B cell maturation antigen (BCMA) for multiple myeloma (MM) [8, 9] and the B cell antigen CD19 for various lymphoid malignancies including B-cell leukemias [10–12] and some types of lymphomas [13, 14]. Indeed, according to published anti-BCMA CAR-T cell clinical trials, complete remission rates of 29 to 60% were reached in a total of 61 patients with relapsed/refractory multiple myeloma (r/r MM) [15]. CAR-T cells targeting CD19 led to initial complete remission in up to 85% of patients with acute lymphoblastic leukemia (ALL) [16] and in up to 100% of patients with refractory or relapsed B cell acute lymphoblastic leukemia (r/r B-ALL) [17]. CAR-T cells targeting large B cell lymphoma are currently approved for second-line therapy after chemotherapy failure [18]. The application of CAR-T cell therapy in hematological malignancies showed promising results that increases the prospect to use this strategy in other types of malignancies.

Currently, there are several ongoing clinical trials utilizing CAR-T cell therapy for solid tumors including glioblastoma [19], lung cancer [20], liver cancer [21], gastric cancer [22], renal cancer [23], prostate cancer [24], osteosarcoma, peritoneal carcinomatosis, pleural cancer, central nervous system tumors and neuroblastoma [25]. This immunotherapeutic approach generated promising clinical outcome. However, it has also shown several radical limitations such as difficulty of the cytotoxic T cells to infiltrate the tumor, insufficiency of T cell recruitment to the tumor site due to abnormal chemokines secreted by solid tumor cells and to the immunosuppressive tumor microenvironment [26, 27]. Moreover, other limitations are related to CAR-T cell side effects including the on-target off-tumor toxicities and the cytokine-released syndrome (CRS) which present the two major adverse events that restrain the therapeutic index [28, 29]. In addition, other toxicities induced by CAR-T cells, such as tumor lysate syndrome, neurotoxicity, cytopenia-related adverse events are also common limitations of this therapy [30]. In the interest of overcoming these obstacles, various innovative strategies are currently under investigation. In addition, scientists are seeking alternative immune effector cells that can be engineered with CARs to be used as antitumor cellular immunotherapy. The increasing understanding of the prominent characteristics of NK cells and macrophages, related to the interaction with other cellular components of the tumor microenvironment, expanded the research focus from CAR-T to CAR-NK and CAR-M cellular immunotherapy [31–35].

Here we discuss the current status, the challenges and prospects regarding the clinical applications of CAR-T, CAR-NK, and CAR-M cells in the management of patients with solid tumors. We also highlight the potential advantages of CAR-NK and CAR-M cells over CAR-T cells.

## CAR-T cell therapy in solid tumors: applications, challenges and recent advances

In recent years, T cells engineered with CAR demonstrated promising outcomes against B cell leukemia and lymphoma, proving its therapeutic anti-cancer potential [36]. Indeed, two CAR-T cell therapies Tisagenlecleucel and Axicabtagen-ciloleucel, were approved by the European Medical Agency (EMA) and the Food and Drug Administration (FDA) for the treatment of patients with relapsed or refractory diffuse large B-cell lymphoma [37–40]. Two additional products have also been approved for these indications: brexucabtagene autoleucel (mantle lymphoma and ALL) and lisocabtagene maraleucel (DBCL, follicular lymphoma, high grade lymphoma). This success is largely due to the choice of the target, the B-cell marker CD19, generating a T cell immune response against the malignant B cells in a MHC-independent manner [41, 42]. Other target antigens: BCMA and CD38 are also found on myeloma cells [37, 38]. Therefore, cellular BCMA-CD38-CAR-T cell therapy is feasible in treating patients with relapsed and refractory multiple myeloma (r/r MM), with high response rate, low recurrence rate and manageable CRS [43]. Importantly, BCMA-CAR-T immunotherapies Ciltacabtagene-autoleucel and Idecabtagene-vicleucel are now available for the treatment of patients with relapsed and refractory multiple myeloma [44]. These significant achievements in the treatment of hematological malignancies advocate CAR-T cell application for the treatment of solid tumors. In recent years, an increasing number of CAR-T cell clinical trials targeting solid tumors have been carried out. In the next subchapter, we report the promising clinical outcomes covering the most common target antigens according to the data provided by ClinicalTrials.gov and the literature.

### Promising clinical outcomes of CAR-T cells in solid tumors

CAR-T cell therapy has achieved important breakthroughs in the treatment of some solid tumors. CAR-T cell clinical trials, targeting several antigens expressed in tumors of different organs, are registered on clinicaltrials.gov and summarized in Table 1. The common CAR-T cell targets in solid tumors have been recently reviewed [45, 46]. Promising clinical outcomes of CAR-T cell therapy in solid tumors are reported in this section according to the targeted tumor antigens.

**Table 1:** Clinical trials of CAR-T cell therapy in solid tumors (ClinicalTrials.gov)

CAR-T product	Clinical trial identifier	Targeted antigen	Disease	Cell source	Clinical trial phase	Status	Estimated enrollment (EE)/ Actual enrollment (AE)/ Treated patients (TP)	Study objectives
MSLN-CAR-T cells	NCT05531708	MSLN	Mesothelin-positive advanced refractory solid tumors	Autologous T cells	Phase 1	Recruiting	20 (EE)	Evaluation of the safety and efficacy of novel mesothelin CAR-T in patients with mesothelin-positive advanced refractory solid tumors.
MSLN-CAR-T cells	NCT05373147	MSLN	Mesothelin-positive solid tumors	Autologous T cells	Early Phase 1	Recruiting	21 (EE)	Evaluation of the safety and tolerability of autologous MSLN-CAR-T cells secreting PD-1 nanobodies (αPD1-MSLN-CAR T cells) in patients with solid tumors.
MSLN CAR-T cells	NCT04489862	MSLN	Non-small cell lung cancer Mesothelioma	Autologous T cells	Early Phase 1	Recruiting	10 (EE)	Evaluation of the safety and tolerability of autologous MSLN-CAR-T cells secreting PD-1 nanobodies (αPD1-MSLN-CAR T cells) in patients with solid tumors.
MSLN CAR-T cells	NCT05141253	MSLN	MSLN-positive solid tumors	RD133 autologous T cells	Early Phase 1	Recruiting	24 (EE)	Evaluation of the safety and efficacy of RD133 MSLN CAR-T cells in subjects with relapsed or refractory MSLN-positive solid tumors.
MSLN CAR-T cells	NCT05166070	MSLN	Solid tumors	RD133 autologous T cells	Early Phase 1	Recruiting	24 (EE)	Evaluation of the Safety and efficacy of RD133 in subjects with relapsed or refractory MSLN-Positive solid tumors.
MSLN CAR T cells secreting PD-1 nanobodies	NCT04489862	MSLN	Non-small-cell lung cancer Mesothelioma	Autologous T cells	Early Phase 1	Recruiting	10 (EE)	Exploratory study of MSLN-CAR-T Cells secreting PD-1 nanobodies for the treatment of MSLN-positive advanced solid tumors.
PD-1 antibody expressing MSLN CAR-T cells	NCT03615313	MSLN	MSLN-positive advanced recurrent or refractory malignant solid tumors	Autologous T cells	Phase 1 Phase 2	Unknown	50 (EE) 1 (TP)	Evaluation of the safety and efficacy of infusion of autologous T cells engineered to target mesothelin and express PD-1 antibodies in adult patients with advanced recurrent or refractory malignant solid tumors, which were positive expression of mesothelin.
MSLN CAR-T cells	NCT02159716	MSLN	Metastatic Pancreatic (Ductal) Adenocarcinoma Epithelial ovarian cancer Malignant epithelial pleural mesothelioma	Autologous T cells	Phase 1	Completed	19 (AE) 15 (TP)	Evaluation of the safety and feasibility of intravenously administered lentiviral transduced CART-meso cells administered with and without cyclophosphamide in a 3 + 3 dose escalation design in patients with metastatic pancreatic cancer, serous epithelial ovarian cancer, or pleural mesothelioma.
αPD1-MSLN-CAR T Cells	NCT04503980	MSLN	Colorectal cancer Ovarian cancer	Autologous T cells	Early Phase 1	Unknown	10 (EE)	Evaluation of the safety and tolerability of autologous mesothelin (MSLN)-targeted chimeric antigen receptor (MSLN-CAR) T cells secreting PD-1 nanobodies (αPD-1-MSLN-CAR T cells) in patients with solid tumors.
CTLA-4/PD-1 antibodies expressing MSLN CAR-T cells	NCT03182803	MSLN	Advanced solid tumors	Autologous T cells	Phase 1 Phase 2	Unknown	40 (EE)	Assessment of the efficacy and safety of the CTLA-4 and PD-1 antibodies expressing mesothelin-CAR-T (mesoCAR-T) for patients with mesothelin positive, advanced recurrent or refractory malignant solid tumors
PD-1 antibody expressing MSLN CAR-T cells	NCT03030001	MSLN	Solid tumors Adult advanced cancer	Autologous T cells	Phase 1 Phase 2	Unknown	40 (EE)	Determination of the safety and efficacy of infusion of autologous T cells engineered to express immune checkpoint antibody and chimeric antigen receptor targeting mesothelin in adult patients with mesothelin positive, recurrent, or refractory malignant tumors.
MSLN CAR-T cells	NCT03545815	MSLN	MSLN-positive multiple solid tumors	Autologous T cells	Phase 1	Unknown	10 (EE)	Evaluation of the feasibility and safety of CRISPR-Cas9 mediated PD-1 and TCR gene-knocked out CAR-T cells in patients with mesothelin positive multiple solid tumors.
GD2/PSMA bi-specific CAR-T cell	NCT05437315	GD2/PSMA	GD2 and PPSMA-positive tumors	Autologous T cells	Phase 1 Phase 2	Recruiting	60 (EE)	Assessment of the feasibility, safety, and efficacy of anti-GD2/PSMA bi-specific CAR-T cell therapy in patients with GD2 and PSMA-positive tumors. Evaluation of the function of the anti-GD2/PSMA bi-specific CAR-T cells and their persistency in patients.
GD2 CAR-T cells	NCT02107963	GD2	Sarcoma Osteosarcoma Neuroblastoma Melanoma	Autologous T cells	Phase 1	Completed	15 (AE)	Evaluation of the antitumor effects, persistence, and safety of GD2 CAR-T cells in children and young adults with GD2-positive solid tumors.
GD2 CAR-T01 cells	NCT03373097	GD2	Neuroblastoma recurrent GD2-positive solid tumors Osteosarcoma Ewing sarcoma	Autologous T cells	Phase 1 Phase 2	Recruiting	42 (EE)	Evaluation of the safety and efficacy of GD2-CART01, a CAR-T cell treatment targeting GD2 in pediatric or young adult patients with high-risk and/or relapsed/refractory neuroblastoma.
GD2 CAR T cells	NCT04196413	GD2	Glioma of spinal cord Glioma of Brainstem	Autologous T cells	Phase 1	Recruiting	45 (EE) 4 (TP)	Evaluating whether GD2-CAR T cells can be successfully made from immune cells collected from children and young adults with H3K27M-mutant diffuse intrinsic pontine glioma (DIPG) or spinal H3K27M-mutant diffuse midline glioma (DMG). H3K27Mmutant testing will occur as part of standard of care prior to enrollment.
GD2 CAR T cells	NCT00085930	GD2	Neuroblastoma	Autologous T cells	Phase 1	Active, not recruiting	19 (TP)	Study of blood T-cells and EBV specific CTLs Expressing GD-2 Specific Chimeric T cell receptors in patients with neuroblastoma
CLDN18.2 CAR-T cells	NCT04467853	CLDN18.2	Claudin18.2-positive advanced solid tumors	LCAR-C18S cells	Phase 1	Recruiting	34 (EE)	Evaluation of the safety, tolerability, pharmacokinetics, and anti-tumor efficacy profiles of the cell-based LCAR-C18S (hereinafter “LCAR-C18S”) in subjects with claudin18.2-positive advanced solid tumors.
CLDN18.2 CAR-T cells	NCT05472857	CLDN18.2	Gastric cancer Pancreatic cancer Advanced ovarian carcinoma Gastroesophageal junction adenocarcinoma	Autologous T cells	Phase 1	Recruiting	30 (EE)	Evaluation of the safety and efficacy of autologous claudin18.2 CAR-T cell therapy in advanced solid tumors with positive CLDN18.2 expression.
CLDN18.2 CAR-T cells	NCT03874897	CLDN18.2	Advanced solid Tumor	Autologous T cells	Phase 1	Recruiting	123 (EE) 37 (TP)	Evaluation of the safety, efficacy, and pharmacokinetics of autologous humanized anti-claudin18.2 CAR-T cells in advanced solid tumor.
CLDN18.2 CAR-T cells	NCT04581473	CLDN18.2	Gastric adenocarcinoma Pancreatic cancer Gastroesophageal junction adenocarcinoma	CT041 autologous T cells	Phase 1b Phase 2	Recruiting	192 (EE)	Evaluation of the efficacy, safety, and pharmacokinetics of CT041 autologous CAR-T cells. Injection in patients with CLDN18.2-positive advanced gastric/ gastroesophageal junction adenocarcinoma and pancreatic cancer.
CLDN18.2 CAR-T cells	NCT05199519	CLDN18.2	Solid tumors	Autologous T cells	Phase 1	Recruiting	30 (EE)	Evaluate the safety, tolerance, pharmacokinetics, and preliminary efficacy of IBI345 in patients with CLDN18.2-positive solid tumors.
CLDN18.2 CAR-T cells	NCT05620732	CLDN18.2	Advanced pancreatic carcinoma Advanced gastric carcinoma	Autologous T cells	N/A	Recruiting	20 (EE)	Evaluation of the efficacy and safety of claudin18.2 CAR-T in advanced pancreatic cancer and gastric carcinoma.
CLDN18.2 CAR-T cells	NCT05472857	CLDN18.2	Gastric cancer Pancreatic cancer Advanced ovarian carcinoma Gastroesophageal junction adenocarcinoma	Autologous T cells	Phase 1	Recruiting	30 (EE)	Evaluation of the safety and efficacy of autologous claudin18.2 CAR-T cell therapy in advanced solid tumors with positive CLDN18.2 expression.
CLDN6 CAR-T cells	NCT04503278	CLDN6	Solid Tumor	Autologous T cells	Phase 1 Phase 2	Recruiting	96 (EE)	Evaluation of safety and preliminary efficacy of CLDN6 CAR-T With or Without CLDN6 RNA-LPX in patients with CLDN6-positive relapsed or refractory advanced solid tumors.
CEA CAR-T cells	NCT05415475	CEA	Colorectal cancer Esophageal cancer Stomach cancer Pancreatic cancer Metastatic tumor Recurrent cancer	Autologous T cells	Phase 1	Recruiting	36 (EE)	Verification of the safety and efficacy of CAR-T cells in the treatment of CEA-positive advanced malignant tumors, and to obtain the recommended dose and infusion scheme of CAR-T cells for the treatment of patients with CEA-positive advanced malignant tumors. Administration method: intravenous infusion or intraperitoneal injection.
CEA CAR-T cells	NCT04348643	CEA	Solid tumor Lung cancer Colorectal cancer Liver cancer Pancreatic cancer Gastric cancer Breast cancer	Autologous T cells	Phase 1 Phase 2	Recruiting	40 (EE)	Evaluation of the efficacy and safety of CEA-targeted CAR-T cells for patients with relapsed/refractory CEA-positive cancer and to obtain the recommended dose and infusion plan.
CEA CAR-T cells	NCT05538195	CEA	Gastric cancer Colon cancer Rectal cancer Esophageal cancer Pancreatic cancer	Autologous T cells	Phase 1 Phase 2	Recruiting	60 (EE)	Evaluation of the safety and efficacy of CEA-targeted CAR-T cell in CEA-positive advanced malignant tumors.
CEA CAR-T cells	NCT05396300	CEA	Colorectal cancer Esophageal cancer Stomach cancer Pancreatic cancer Metastatic tumors Recurrent cancer	Autologous T cells	Phase 1	Completed	60 (EE)	Evaluation of the safety and tolerability of CAR-T in patients with CEA-positive advanced malignant solid tumors, and to obtain the maximum tolerated dose of CAR-T and phase II recommended dose.
CEA CAR-T cells	NCT02349724	CEA	Lung cancer Colorectal cancer Gastric Cancer Breast Cancer Pancreatic Cancer	Autologous T cells	Phase 1	Unknown	75 (EE) 10 (TP)	Verification of the safety of CEA targeted chimeric antigen receptor T cells and to determine the proper dosage of CAR T cells infused
CEA CAR-T cells	NCT02416466	CEA	Liver metastases	Autologous T cells	Phase 1	Completed	8 (AE) 6 (TP)	Study of anti-CEA CAR-T cells hepatic artery infusions and yttrium-90 SIR-Spheres in patients with CEA-expressing liver metastases
CEA CAR-T cells	NCT02850536	CEA	Liver metastases	Autologous T cells	Phase 1b	Completed	5 (AE) 1 (TP)	Study of anti-CEA CAR-T cell infusions delivered via the hepatic artery or splenic vein using the Sure-fire Infusion System (SIS) for patients with CEA-expressing liver metastases or pancreas cancer.
ROR1 CAR-T cells	NCT05274451	ROR1	Triple-negative breast cancer Non-small cell lung cancer Metastatic non-small cell carcinoma of the lung Breast cancer Advanced lung carcinoma Recurrent NSCLC Relapse/recurrence breast cancer	LYL797 autologous T cells	Phase 1	Recruiting	54 (EE)	Assessment of the safety and efficacy of LYL797, ROR1-targeting CAR-T cells, in adults with relapsed and/or refractory solid-tumor malignancies.
ROR1 CAR-T cells	NCT02706392	ROR1	Advanced ROR1-positive TNBC and NSCLC	Autologous T cells	Phase 1	Terminated	21 (AE) 5 (TP)	Evaluation of the safety and anti-tumor activity of adoptively transferred autologous ROR1 CAR-T cells in pts. with advanced ROR1+ TNBC and NSCLC.
ROR2 CAR-T cells	NCT03960060	ROR2	Recurrent or refractory solid tumors	CCT301–59 autologous T cells		Active, not recruiting	18 (EE)	Evaluation of the safety and preliminary therapeutic efficacy of CCT301–59 T cells in adult subjects with relapsed and refractory stage IV metastatic solid tumors (soft tissue sarcoma, gastric cancer, pancreatic cancer, bladder cancer).
NKG2DL CAR-T cells	NCT05382377	NKG2DL	CRC, advanced NKG2DL-positive solid tumors	KD-025 autologous cell	Phase 1	Recruiting	18 (EE)	Evaluation of the safety and effectiveness of NKG2D-based CAR-T cell infusion in the treatment of advanced NKG2DL-positive solid tumors.
NKG2DL+/CLDN18.2+ CAR-T cells	NCT05583201	NKG2D/CLDN18.2	NKG2D/CLDN18.2-positive solid tumor	KD-496 autologous cells	Phase 1	Recruiting	18 (EE)	Evaluation of the safety and effectiveness of NKG2D/CLDN18.2-based CAR-T cell infusion in the treatment of advanced NKG2DL+/CLDN18.2+ solid tumors.
NKG2DL CAR-T cells	NCT04107142	NKG2DL	Colorectal Cancer Triple negative breast cancer Sarcoma Nasopharyngeal carcinoma Prostate cancer Gastric cancer	Haploidentical / Allogeneic T cells	Phase 1	Unknown	10 (EE)	Evaluation of the haploidentical / allogeneic Natural Killer Group 2D Ligand (NKG2DL)-targeting chimeric antigen receptor-grafted Gamma Delta (γδ) T Cells (CTM-N2D) in subjects with relapsed or refractory solid tumour
CD70 CAR-T cells	NCT05420545	CD70	Metastatic tumor Advanced solid tumor Renal cell carcinoma Ovarian cancer Cervix cancer	Autologous T cells	Phase 1	Recruiting	36 (EE)	Evaluation of the safety and tolerability of CAR-T in patients with CD70-positive advanced/metastatic solid tumors, and to obtain the maximum tolerated dose of CAR-T and phase II recommended dose.
CD276 CAR-T cells	NCT04691713	CD276	CD276-positive advanced solid tumors	Autologous T cells	N/A	Unknown	5 (EE)	Evaluation of the safety and effectiveness of targeting CD276 auto-chimeric antigen receptor T cells in the treatment of CD276-positive advanced solid tumors.
HER2 CAR-T cells	NCT04650451	HER2	HER2-positive gastric cancer HER2-positive breast cancer HER-2 protein overexpression solid tumor	BPX-603 autologous T cells	Phase 1 Phase 2	Recruiting	220 (EE)	Investigation of the safety, tolerability, and clinical activity of HER2-specific dual-switch CAR-T cells, BPX-603, administered with rimiducid to subjects with previously treated, locally advanced or metastatic solid tumors which are HER2 amplified/overexpressed.
HER2 CAR-T cells	NCT04511871	HER2	Solid tumor Gastric cancer Breast cancer Ovarian cancer Sarcoma	Autologous T cells	Phase 1	Recruiting	15 (EE)	Assessment of the safety, tolerability, and anti-tumor activity of autologous CAR-T cells (CCT303–406) in patients with relapsed or refractory HER2 Positive solid tumors.
HER2 CAR-T cells	NCT03740256	HER2	Solid tumors	Autologous T cells	Phase 1	Recruiting	45 (EE)	Study of the effect of binary oncolytic adenovirus in combination with HER2-Specific autologous CAR-T Cells in patients with advanced HER2 Positive solid tumors.
HER2 CAR-T cells	NCT00902044	HER2	Advanced sarcoma	Autologous T cells	Phase 1	Active, not recruiting	36 (EE) 19 (TP)	Administration of HER2 CAR-T cells for subjects with advanced sarcoma (HEROS)
HER2 CAR-T cells	NCT01109095	HER2	Glioblastoma multiforme	Autologous T cells	Phase 1	Completed	16 (AE) 17 (TP)	Evaluation of the safety and antitumor efficacy of autologous HER2-specific chimeric antigen receptor (CAR)–modified virus-specific T cells (VSTs) in patients with progressive glioblastoma
HER2 CAR-T cells	NCT01935843	HER2	Advanced HER-2-positive solid tumors	Autologous T cells	Phase 1 Phase 2	Unknown	10 (EE) 11 (TP)	Evaluation of the safety, feasibility, and activity of CAR-T cell immunotherapy targeting human epidermal growth factor receptor 2 (HER2) in patients with advanced biliary tract cancers (BTCs) and pancreatic cancers (PCs)
HER2 CAR-T cells	NCT03500991	HER2	HER2-positive recurrent/refractory pediatric CNS tumors	Autologous T cells	Phase 1	Recruiting	48 (EE) 3 (TP)	Assessing the feasibility, safety, and tolerability; secondary objectives include assessing CAR-T cell distribution and disease response
EGFR-TGFβR-KO CAR-T cells	NCT04976218	EGFR	EGFR-positive solid tumors	Autologous T cells	Phase 1	Recruiting	30 (EE)	Evaluation of the anti-tumor activities and safety profiles of CAR-EGFR-TGFβR-KO T cell in previously treated advanced EGFR positive solid tumors. CAR-EGFR-TGFβR-KO T cell engineered by knocking out TGF-β receptor II through CRISPR/Cas9.
Anti-CTLA-4/PD-1 expressing EGFR-CAR-T	NCT03182816	EGFR	EGFR-positive advanced solid tumors	Autologous T cells	Phase 1 Phase 2	Unknown	40 (EE) 9 (TP)	Assessment of the efficacy and safety of the CTLA-4 and PD-1 antibodies expressing EGFR-CAR-T for patients with EGFR positive advanced recurrent or refractory malignant solid tumors
EGFR CAR-T cells	NCT01869166	EGFR	EGFR-positive advanced solid tumors	Autologous T cells	Phase 1 Phase 2	Unknown	60 (EE) 14 (TP)	Evaluation of the safety, feasibility of the chimeric antigen receptor T cells transduced with the anti-EGFR and their in vivo survival duration.
EGFRvIII CAR-T cells	NCT02209376	EGFRvIII	Residual or reccurent EGFRvIII- positive Glioma	Autologous T cells	Phase 1	Terminated	11 (EE) 10 (TP)	Evaluation of the safety and feasibility of CART-EGFRvIII (autologous T cells transduced with a lentiviral vector to express a chimeric antigen receptor specific for EGFRvIII) in the treatment of patients with EGFRvIII+ glioblastoma who have had their first recurrence as determined by standard imaging or have residual disease after initial resection.
PD-1 antibody expressing EGFR CAR-T Cells	NCT02873390	EGFR	EGFR-positive advanced solid tumors	Autologous T cells	Phase 1 Phase 2	Unknown	20 (EE)	Evaluation of the safety and effectiveness of cell therapy using herinCAR-PD1 cells to treat advanced cancer.
PD-1 antibodies expressing EGFR CAR-T Cells	NCT02862028	EGFR	EGFR-positive advanced solid tumors (Lung, Liver and Stomach)		Phase 1 Phase 2	Unknown	20 (EE)	Evaluation of the safety and effectiveness of cell therapy using herinCAR-PD1 cells to treat relapsed or refractory cancer
VEGFR1/PD-L1 CAR-T cells	NCT05477927	VEGFR1 and PD-L1	Malignant peritoneal effusion Malignant ascites Serous cavity metastatises	Autologous T cells	Phase 1	Recruiting	58 (EE)	Dose-escalation and expansion study of specific dual-targeting VEGFR1 and PD-L1 CAR-T in cancer patients with pleural or peritoneal metastases.
EGFR/B7-H3 CAR-T cells	NCT05341492	EGFR and B7-H3	Advanced lung cancer Advanced triple-negative breast cancer	Autologous T cells	Early Phase 1	Recruiting	30 (EE)	Evaluation of the safety and efficacy of EGFR/B7H3 CAR-T in patients with EGFR/ B7-H3-positive advanced solid tumors (Lung and Triple-negative Breast Cancer).
B7-H3 CAR-T cells	NCT04897321	B7-H3	Pediatric solid tumor Sarcoma	Autologous T cells	Phase 1	Recruiting	32 (EE)	Evaluation of the use of autologous T cells genetically engineered to express B7-H3-CARs for patients ≤21 years old, with relapsed/refractory B7-H3-positive solid tumors. Evaluation of the safety and maximum tolerated dose of B7-H3-CAR-T cells. Finding the highest dose of B7-H3-CAR T cells that are safe to give to patients with B7-H3-positive solid tumors.
B7-H3 CAR-T cells	NCT04483778	B7-H3	Recurrent/Refractory solid tumors in children and young adults	Allogeneic T cells	Phase 1	Recruiting	68 (EE)	Evaluation of the safety, feasibility, and efficacy of administering T cell products derived from the research participant’s blood that have been genetically modified to express a B7-H3-specific receptor CAR that will target and kill solid tumors that express B7-H3.
B7-H3 CAR-T cells	NCT05190185	B7-H3	Malignant melanoma lung cancer, or colorectal cancer	TAA06 autologous T cells	Phase 1	Recruiting	18 (EE)	Evaluation of the safety and tolerability of TAA06 CAR-T cells targeting B7-H3 in patients with advanced solid tumors. Evaluation of the distribution, proliferation, and persistence of B7-H3-targeted CAR T cells and their efficacy.
CD276 CAR-T	NCT04691713	CD276	Solid tumors	Autologous T cells	N/A	Recruiting	5 (EE)	Evaluation of the safety and effectiveness of targeting CD276 auto-CAR-T cells in the treatment of CD276-positive advanced solid tumors.
CTLA-4 and PD-1 antibodies expressing MUC1-CAR-T Cells	NCT03179007	MUC1	MUC1-positive advanced recurrent or refractory malignant solid tumors.	Autologous T cells	Phase 1 Phase 2	Unknown	40 (EE)	Evaluation of the safety and efficacy of infusion of autologous T cells engineered to express immune checkpoint antibodies (CTLA-4 and PD-1) and chimeric antigen receptor targeting MUC1 in adult patients with MUC1 positive, advanced recurrent or refractory malignant solid tumors.
PD-1 -Knockout MUC1 CAR-T cells	NCT03706326	MUC1	Advanced esophageal cancer	Autologous T cells	Phase 1 Phase 2	Unknown	20 (EE)	Assessment of the safety and efficacy of the immunotherapies using anti-MUC1 CAR T cells and /or PD-1 knockout engineered T cells in the treatment of patients with advanced esophageal cancer.
MUC1 CAR T Cells	NCT02587689	MUC1	Hepatocellular carcinoma Non-small Cell Lung Cancer Pancreatic Carcinoma Triple-negative invasive breast carcinoma	Autologous T cells	Phase 1 Phase 2	Unknown	20 (EE)	Determination of whether autologous T cells bearing chimeric antigen receptor that can specifically recognize (Mucin 1) MUC1 is safe and effective for patients with relapsed or refractory solid tumor.
MUC1 CAR T Cells	NCT02617134	MUC1	Malignant glioma of brain Colorectal carcinoma Gastric Carcinoma	Autologous T cells	Phase 1 Phase 2	Unknown	20 (EE)	Evaluation of the safety and effectiveness of CAR-T cell immunotherapy in patients with MUC1 positive relapsed or refractory solid tumors.
P-MUC1C-ALLO1 CAR-T cells	NCT05239143	MUC1C	Breast cancer Ovarian cancer Non-small cell lung cancer Colorectal cancer Pancreatic cancer Renal cell carcinoma Nasopharyngeal cancer Head and neck squamous cell carcinoma Gastric cancer	Allogeneic T cells	Phase 1	Recruiting	100 (EE) 3 (TP)	Determination of the recommended phase 2 dose of P-MUC1C-ALLO1 an allogeneic CAR-T cell therapy designed to target cancer cells expressing Mucin1 cell surface-associated C-Terminal (MUC1-C) antigen.
TnMUC1 CAR-T cells	NCT04025216	TnMUC1	Advanced TnMUC1-positive solid tumors	Autologous T cells	Phase 1	Active, not recruiting	112 (EE)	Identification of the dose and regimen of CART-TnMUC1 cells that can be safely administered intravenously following the lymphodepletion (LD) regimen to patients with (1) advanced TnMUC1+ solid tumors (triple negative breast cancer, epithelial ovarian cancer, pancreatic cancer, and non-small cell lung cancer) and (2) advanced TnMUC1+ multiple myeloma
Lewis Y CAR-T cells	NCT03851146	Lewis Y	Advanced cancer	Autologous T cells	Phase 1	Completed	20 (EE)	Investigation of the safety, tolerability, and immunological effects of T Lymphocytes transduced with an anti-Lewis Y (LeY) CAR gene (LeY-CAR-T) in patients with LeY antigen expressing advanced solid tumors.
OX40 CAR-T cells	NCT04952272	OX40	Lung cancer Hepatocellular carcinoma Solid tumor	Autologous T cells	Phase 1	Recruiting	50 (EE)	Evaluation of the safety and clinical effects of intratumor injecting CpG-ODN and in situ release of tumor antigen by interventional ablation or drug-eluting beads to treat advanced solid tumors. With or without infusion of CAR-T cells secreting scFv against OX40.
EpCAM CAR-T cells	NCT02915445	EpCAM	Malignant neoplasm of nasopharynx TNM staging distant metastasis Breast cancer Recurrent gastric cancer with metastasis	Autologous T cells	Phase 1	Recruiting	30 (EE)	Determination of the safety of CAR-T cells recognizing EpCAM.
TM4SF1- and EpCAM- CAR-T cells	NCT04151186	TM4SF1/ EpCAM	EpCAM-positive Recurrent/Refractory solid tumors	Autologous T cells	N/A	Unknown	72 (EE)	Evaluation of the Safety and efficacy of CAR-T-cell therapy for the TM4SF1- and EpCAM-positive Recurrent/Refractory solid tumors
NKG2DL CAR-T cells	NCT05382377	NKG2DL	Solid tumors	KD-025 autologous T cells	Early Phase 1	Recruiting	30 (EE)	Evaluation of the safety and efficacy of KD-025 CAR-T Therapy in advanced NKG2DL-positive solid tumors.
PSCA CAR-T cells	NCT02744287	PSCA	Metastatic Castration-resistant Prostate cancer Metastatic prostate cancer	BPX-601 autologous T cells	Phase 1 Phase 2	Recruiting	151 (EE)	Evaluation of the feasibility, safety, and activity of PSCA-Specific CAR Engineered T Cells (BPX-601) in subjects with previously treated advanced solid tumors.
GUCY2C CAR-T cells	NCT05287165	GUCY2C	Advanced solid tumors Digestive system neoplasms Pancreatic cancer Resectable colorectal (colon or rectal) cancer	IM96 autologous T cells	Early Phase 1	Recruiting	19 (EE)	Evaluation of the safety and efficacy of IM96 CAR-T Cells therapy in patients with advanced digestive system neoplasms.
4SCAR-IgT- cells	NCT03356782	CD133, GD2, MUC1, CD117	Sarcoma osteoid Sarcoma Ewing Sarcoma	Autologous T cells	Phase1 Phase 2	Recruiting	20 (EE)	Evaluation of the safety and efficacy of 4th generation 4SCAR-IgT cells targeting sarcomas.
IL7, CCL19, IL12-expressing Nectin4 CAR-T cells	NCT03932565	Nectin4	Nectin4-positive malignant solid tumors	Autologous T cells	Phase 1	Unknown	30 (EE)	Study of the intravenous minimally invasive surgery combined with intratumoral injection of Nectin4/FAP-targeted fourth-generation CAR-T cells (expressing IL7 and CCL19, or IL12) are used to treat Nectin4-positive advanced malignant solid tumors, maximally eliminating residual cancer cells, and preventing recurrence.
GPC3 CAR-T cells	NCT02932956	Glypican 3	Pediatric solid tumors	Autologous T cells	Phase 1	Active, not recruiting	10 (EE)	The purpose of this study is to find the biggest dose of GAP T cells that is safe, to see how long they last in the body, to learn what the side effects are and to see if the GAP T cells will help people with GPC3-positive solid tumors. This study enrolls patients who have GPC3-positive solid tumors
CAR-T cells	NCT03356795	GD2, PSMA, MUC1, MSLN or other markers	Cervical cancer	Autologous T cells	Phase 1 Phase 2	Unknown	20 (EE)	Assessment of the feasibility, safety, and efficacy of CAR T cells immunotherapy in patients who have GD2, PSMA, Muc1, Mesothelin or other markers positive cervical cancer. Another goal of the study is to learn more about the persistence and function of CAR T cells in the body.
CAR-T cells	NCT04981119	N/A	Solid tumor Colorectal cancer Non-small cell lung cancer Pancreatic cancer	Autologous T cells	N/A	Recruiting	200 (EE)	Collect information on how often a solid tumor cancer might lose the Human Leukocyte Antigen (HLA) by next-generation sequencing and perform leukapheresis to collect and store an eligible participant’s own T cells for future use to make CAR-T Cell therapy for their disease treatment.
N/A	NCT04082910	N/A	Solid tumor Hematological malignancy	Autologous T cells	Phase 1 Phase 2	Recruiting	30 (EE)	Evaluation of the feasibility and efficacy of metoprolol, a beta-1 adrenergic receptor blocker, in the treatment of cytokine release syndrome (CRS) caused by CAR-T cell infusions. Evaluation of the effects of the treatment on the serum levels of Interleukin-6 (IL-6) and other cytokines.

#### Clinical outcomes of CAR-T cell therapy targeting HER2

Encouraging outcomes were demonstrated in a phase I/II clinical study (NCT00902044) using human epidermal growth factor receptor-2 (HER2)-CAR-T cells in the treatment of 19 patients with HER2-positive sarcomas (16 osteosarcomas, 1 primitive neuroectodermal, 1 Ewing sarcoma, and 1 protofibroblastic small round cell tumor) [39]. In this study, out of 17 evaluable patients, 4 experienced stable disease for 3 to 14 months, 3 of these patients received no additional therapy and had their tumor removed, with 1 showing ≥90% tumor necrosis [47]. The 19 infused patients had a median overall survival of 10.3 months (from 5.1 to 29.1 months). Interestingly, no adverse events were observed after high-dose treatment of CAR-T cell, except for high fever in one patient [39]. Moreover, a phase I study (NCT01109095) of a HER2 specific CAR-T cell treatment of 17 patients with glioblastoma reported a great tolerance to the CAR-T cells administered doses and a median overall survival (OS) of 11.1 months for 8 patients after treatment and 24.5 months after diagnosis. Furthermore, 3 patients were alive with no disease progression at the last follow-up [19]. A phase I clinical trial (NCT01935843) of CAR-T cells targeting the HER2 marker in 11 patients with pancreatic cancers (PCs) and advanced biliary tract cancers (BTCs) observed a median OS of 4.8 months (range, 1.5–8.3 months) with minimal and reversible toxic effects [48]. An additional phase I clinical trial (NCT03500991) conducted in a group of young people and children with refractory or recurrent Central Nervous System (CNS) tumors, including diffuse midline gliomas, demonstrated that iterative local HER2 CAR- T cell administration induced increased secretion of chemokines like C-C Motif Chemokine Ligand 2 (CCL2) and C-X-C motif chemokine ligand 10 (CXCL10) in the cerebrospinal fluid with no CAR-T cell dose-related toxicity [49]. These findings allow the suggestion of developing CXCR/CCR-expressing-CAR-T cells thereby improving their binding with the tumor antigen.

#### Clinical outcomes of CAR-T cell therapy targeting IL-13Rα2

IL-13Rα2 is highly expressed in glioblastoma (GBM) tumor cells but is rarely expressed in normal brain cells, making it an interesting target for CAR-T cell therapy in glioblastoma cancer [40].. In Brown and colleagues’ study (NCT02208362), multi-dose treatment with IL-13Rα2-CAR-T cells induced a complete tumor regression for nearly 8 months in a patient with disseminated glioblastoma [40]. For the same targeted tumor antigen, another clinical trial (NCT00730613) used anti-IL-13Rα2-CAR-T cells for the treatment of 3 patients with recurrent GBM [50]. The therapy was well tolerated with controlled brain inflammation in all patients with recurrent disease. A short remission was observed in one patient, possibly due to IL-13Rα2 antigen loss on the relapsing tumor [50].

#### Clinical outcomes of CAR-T cell therapy targeting GD2

In neuroblastoma cells, disialoganglioside (GD2) is highly expressed [51] and might be considered as another interesting target for CAR-T cells in GBM. Recently, Majzner et al. [52], reported the outcomes of a first-in-human phase I clinical trial (NCT04196413) in 4 patients with H3K27M-mutated DIPG or spinal cord DMG-treated with GD2 CAR-T cells. Three out of four patients exhibited clinical and radiographic improvement associated with high levels of pro-inflammatory cytokines in the plasma and cerebrospinal fluid without on-target/off-tumor “OTOT” toxicity. Furthermore, a phase-I clinical trial (NCT00085930) evaluating GD2 CAR-T cells effect on 11 patients with neuroblastoma, showed complete remission in 3 patients [53]. GD2 was also targeted in a phase-I trial (ACTRN12613000198729) for GD2 positive metastatic melanoma patients treated with CAR-T cell therapy. The data of this study showed upregulated lymphocyte-activation gene 3 (LAG-3) and programmed cell death protein1 (PD-1) expression in administered CAR-T cells [54]. Therefore, combined CAR-T cells with PD-1 immune checkpoint blockade may enhance the efficacy of CAR-T cell therapy.

#### Clinical outcomes of CAR-T cell therapy targeting ROR1

The orphan tyrosine kinase receptor ROR1 is a candidate target for CAR-T cell therapy because it is expressed on the surface of many lymphatic and epithelial malignancies and has a putative role in tumor cell survival [55]. A phase I trial (NCT02706392) examined the efficacy and safety of CAR-T cell targeting the transmembrane tyrosine kinase receptor (ROR1) expressed in lung and breast cancers [56]. In this study, 4 out of 5 patients with lung and breast cancer experienced a mixed response with lower tumor burden at some metastatic sites [56].

#### Clinical outcomes of CAR-T cell therapy targeting EGFR

The epidermal growth factor receptor (EGFR) plays an important role in the development and progression of solid tumors and has emerged as an important therapeutic target in different types of cancer such as non-small-cell lung carcinoma, breast, gastroesophageal and colorectal cancers [57]. In addition, many clinical trials have been conducted on CAR-T cells targeting EGFR for the treatment of EGFR-positive solid tumors [45]. A phase-I clinical trial (NCT01869166) of EGFR CAR-T cell therapy in 11 patients with EGFR+ refractory/ relapsed non-small cell lung cancer (NSCLC) showed that 2 patients achieved partial response and 5 had stable disease for 2 to 8 months without severe toxicity [58]. Moreover, in a phase-I clinical trial, 10 patients with recurrent EGFRvIII+ glioblastoma (GBM) were treated with EGFRvIII engineered CAR-T cells (NCT02209376) [59]. The results of this study showed an anti-tumor effect of CAR-T cells with a median OS of approximately 8 months in all patients [59]. Other antigens that are targeted by CAR-T cells for GBM therapy are ephrin type-A receptor 2 (EphA2) (NCT02575261), and mucin 1 (MUC1) (NCT02839954, NCT02617134). In addition, in a phase I clinical trial (NCT03182816), EGFR was targeted by non-viral piggyBac transposon system-engineered EGFR-CAR T-cell therapy in 9 patients with non-small cell lung cancer. In this study, 1 patient had a sustained response of more than 13 months while 6 had stable disease, and 2 had progressed disease. The median progression-free survival was 7.13 months, with a median overall survival of 15.63 months [60]. This study showed that the non-viral piggyBac transposon system-engineered EGFR-CAR T-cell therapy is feasible and safe in the treatment of EGFR-positive advanced relapsed/refractory NSCLC patients [60].

#### Clinical outcomes of CAR-T cell therapy targeting CEA

A high level of Carcinoembryonic antigen (CEA) is known to be associated with poor cancer prognosis [61]. For this reason CEA has been targeted for the treatment of lung [62], breast [63], pancreatic and gastric cancers [64, 65], and it is considered as one of the most promising targets for colorectal cancer (CRC) [66]. A phase-I, escalating-dose trial of CAR-T cell therapy (NCT02349724) targeting CEA expressed in metastatic CRC reported that 7 out of 10 patients presented stable disease for up to 30 weeks and 2 patients experienced tumor reduction with no adverse events [67]. Additionally, Katz et al. [68] tested the effect of intra-arterial anti-CEA CAR-T cells therapy combined with internal radiation therapy in 6 patients with liver metastases. The data of this phase-Ib clinical trial (NCT02416466) demonstrated tolerated response to CAR-T cell therapy with non-observed grade 4 or 5 toxicities, and without instances of severe CRS or neurotoxicity. Additionally, the median survival OS was 8 months [68]. Moreover, anti-CEA CAR-T cell therapy demonstrated a significant beneficial effect in a patient with liver metastases secondary to stage IV pancreatic adenocarcinoma, and who received locally infused CEA CAR-T cells at the site of a solid tumor by Pressure-enabled Drug Delivery (PEDD) [69]. In this clinical trial (NCT02850536), anti-CEA CAR-T cell induced a complete metabolic response within the liver which was durable and sustained for 13 months with no serious adverse events above grade 3, highlighting the importance of combining CAR-T cell therapy with PEDD technology [69].

#### Clinical outcomes of CAR-T cell therapy targeting MSLN

Several CAR-T cell clinical trials targeting Mesothelin^+^ (MSLN) ovarian cancer (OC) have been undertaken. In a phase I/II clinical study (NCT03615313), a patient with relapsed epithelial OC was treated with MSLN-CAR-T cells and PD-1 blockade in combination with lapatinib, an angiogenesis inhibitor [70]. Interestingly, the patient achieved partial remission, survived for more than 17 months and experienced minimal side effects like grade-1 hypertension and fatigue [70]. Moreover, CAR-T cells targeting MSLN were administered to patients with pancreatic ductal adenocarcinoma, malignant pleural mesothelioma and OC who participated in a phase I clinical trial (NCT02159716) [71]. The study showed that lentiviral-transduced MSLN CAR-T cells expanded well in the peripheral blood, but their persistence was limited despite pre-treatment with cyclophosphamide [71]. Post-treatment stable disease in over 11 patients out of 15, was the best overall response reported in this study [71]. In another study, the intravenous injection of interleukin 7 (IL-7) and Chemokine (C-C motif) ligand-19 (CCL19)-MSLN secreting CAR-T cells (NCT03198546) in a patient with advanced pancreatic cancer induced complete regression of the tumor 240 days post-treatment [72]. No high-grade adverse events were observed [72].

#### Clinical outcomes of CAR-T cell therapy targeting CD133

CD133 is widely used as a marker to identify CRC stem cells and endothelial progenitor cells [73]. It can also be used to predict tumor progression, patient survival and chemoradiotherapy resistance in CRC [74, 75] and is one of the most well-characterized markers of cancer stem cells (CSCs) in various tumor types, including hepatocellular carcinoma (HCC) [76]. In a phase I/II clinical trial (NCT02541370), CD133 CAR-T cells were administered to 21 patients with advanced hepatocellular carcinoma [77]. This study demonstrated antitumor efficacy with low treatment-related toxicity. Of 21 evaluable patients, 1 had a partial response, 14 had stable disease for 2 to 16.3 months, and 6 had progressed disease after CAR-T cell administration [77]. Only 4 patients developed grade 3 hyperbilirubinemia and 2 patients had grade-3 anemia with no other serious adverse events [77].

#### Clinical outcomes of CAR-T cell therapy targeting Claudin 18.2

Claudin18.2, a stomach-specific isoform of Claudin-18, is expressed in 70% of primary gastric adenocarcinomas and their metastases [78]. It is considered as a potential target for the treatment of these malignancies. CT041, an anti-CLDN18.2 CAR-T cell product, has received Investigational New Drug (IND) clearance from the United States FDA in patients with CLDN18.2-expressing stomach, pancreatic, and gastroesophageal junction adenocarcinoma [79]. The IND clearance was supported by a phase-I trial (NCT03874897) which found that a Claudin18.2 CAR-T cell resulted in an overall response rate and disease control rate of 57.1 and 75.0%, respectively, in gastric cancer patients and the 6-month overall survival rate was 81.2%. No serious adverse events were reported [80]. This CAR-T cell therapy study resulted in an overall response rate (ORR) of 33%, a median progression free survival (PFS) of 130 days and a tolerable safety profile with no serious adverse events [80].

#### Clinical outcomes of CAR-T cell therapy targeting MUC1

Glycoprotein Mucin 1 (MUC1) is a transmembrane protein that belongs to the mucin family. This molecule is associated with metastases and tumor progression, especially in stomach cancer [81]. An open-label dose-escalation phase-I study (NCT05239143) has been recently activated to study the treatment effects of P-MUC1C-ALLO1 in subjects with advanced or metastatic solid tumors [82]. P-MUC1C-ALLO1 is an allogeneic CAR-T cell therapy designed to target cancer cells expressing Mucin 1 cell surface-associated C-terminal antigen. The results of this study showed an early efficacy at the low dose of the CAR-T cells with partial response in one patient (HR+, Her2- Breast cancer). No P-MUC1C-ALLO1 related toxicities were observed [82]. Although CAR-T cell therapy showed encouraging clinical outcomes, a meta-analysis on the efficacy of this innovative approach on solid tumors, showed a comprehensive response rate of only 9% [29]. Indeed, various drawbacks hinder the efficacy of CAR-T cell therapy against solid tumors including the lack of tumor-specific antigens (TSAs) and antigen heterogeneity [83]. Moreover, CAR-T cell trafficking and infiltration in the tumor site [84] and the immunosuppressive tumor microenvironment are major limitations, significantly impeding the function and persistence of CAR-T cells [85–87]. Due to these challenges, improving the design of CAR-T cell therapy for solid tumors merits special considerations in the future.

### Challenges of CAR-T cells and innovative strategies to advance this therapy for solid tumors

Major challenges for CAR-T cell therapy in solid tumors include the identification of truly specific tumor antigens as targets, overcoming tumor antigen escape, improving CAR-T cells trafficking, infiltration and expansion at the tumor site as well as their persistence and functions in a hostile tumor microenvironment. To overcome these challenges and to enhance efficiency of CAR-T cells in solid tumors, various strategies have been developed such as optimizing CAR constructs or identifying innovative therapeutic combination strategies, thereby enhancing specificity, infiltration, and efficacy of CAR-T cell treatment and to modulate the inhibitory conditions (Table 2).

**Table 2:** Advantages, limitations, and potential strategies improving CAR-T, CAR-NK and CAR-M therapy

	CAR-T cells	CAR-NK cells	CAR-M cells
**Advantages**	­- Sufficient number of circulating T cells -­ Previous studies on hematological malignancies facilitating its use on solid tumors	­- Natural ability against non-self-cells ­- Direct and indirect killing functions due respectively to CAR and ADCC ­- Self-identification of normal cells by KIR -­ Reduced risk of CRS, ICANS and GvHD -­ Can be generated from different sources	-­ M1 macrophages feature a pro-inflammatory phenotype ­- Antitumor activity by phagocytosis, presenting tumor antigen to Th1 cells and production of anti-inflammatory factors -­ Most abundant population in the TME of many cancer types -­ Important source of matrix metalloproteinase (MMP) which degrades almost all ECM ­- Can be generated from different sources
**Limitations**	­- Tumor antigen heterogeneity and tumor antigens loss -­ Difficulty in infiltrating tumors -­ Limited survival and persistence in the immunosuppressive tumor microenvironment -­ CRS, OTOT toxicity, neurotoxicity and GvHD	-­ Limited tumor infiltration -­ Limited efficacy in CAR transduction -­ Limited survival and persistence in the immunosuppressive tumor microenvironment	-­ Limited efficacy in CAR transduction -­ CRS toxicity -­ OTOT toxicity -­ Need differentiation to M1 phenotype
**Strategies**	➣ **Overcoming tumor antigen heterogeneity and tumor antigen loss:** -­ Bispecific-CAR-T cells -­ Pooled CAR-T cells -­ Switch on or off CAR-T cells -­ AI (radiomics) ➣ **Facilitating CAR-T cell tumor infiltration:** -­ Nanobody-based CAR-T cell therapy -­ Chemokine receptor-expressing CAR-T cells -­ CAR-T cells local administration: intraperitoneal, intra-tumoral injection, porous microneedle patch -­ CAR-T cells targeting stromal cell-associated antigens -­ Matrix-degrading enzymes-secreting CAR-T cells -­ Molecular torpedo -­ Modifying CARs design, e.g., Hinge domain, transmembrane domain and co-stimulatory signaling -­ Alternative non-LV or RV transduction and in vivo delivery of CARs -­ CAR-T cells combination with ICIs (anti-CTLA-4 or anti-PD-1 monoclonal antibodies) -­ PD-1/CTLA-4- antibodies secreting CAR-T cells ➣ **Overcoming the immunosuppressive tumor microenvironment and persistence:** -­ CAR-T cells secreting immunostimulatory factors such as IL12, IL18, and IL15 -­ CAR-T cells targeting Treg, MDSCs and M2 macrophages -­ Combining CAR-T cells with chemotherapy ➣ **Overcoming CAR-T cells’ CRS toxicity:** -­ IL-1R antagonists-secreting CAR-T -­ IL-6 blockade -­ Neutralizing GM administration -­ CAR construct improvement -­ Control of CAR activity and survival in vivo	➣ **Improving the trafficking to the tumor site:** -­ CAR-NK expressing chemokine receptors ➣ **Improving the transduction efficiency of NK cells:** -­ Retronectin, ectofusin-1 used as transduction enhancer -­ Baboon envelope pseudotyped lentivirus (BaEV-LV) -­ Electroporation and transposons for non-viral transduction ➣ **Improving CAR-NK cytotoxicity:** -­ Armored CAR-NK with co-stimulatory domains (DAP-10, DAP-12 or 2B4) -­ Combining CAR-NK with tyrosine kinase inhibitors -­ Combining CAR-NK with immune checkpoints inhibitors (anti-PD-1 antibodies) ➣ **Improving in vivo survival and persistence within the TME:** -­ Engineered CAR-NK to co-express stimulatory cytokine -­ Designed chimeric co-stimulatory converting receptor (CCCR)-NK for switching the immunosuppressive negative signal to an activating one -­ Combining CAR-NK cells with chemotherapy and radiotherapy ➣ **Improving NK cell generation:** -­ Using different sources of NK cells including NK92 cell line, iPSCs, hESC.	➣ **Improving the bioengineering of CAR-M:** -­ Use of modified lentiviral virions containing Vpx -­ Use of adenovirus 5-fiber 35 vector (Ad5f35) for efficient gene transfer -­ Mannose-conjugated polyethyleneimine (MPEI) for effective gene delivery ➣ **Enhancing the antitumor activity of CAR-M:** -­ M2 to an M1 phenotype polarization -­ CAR iMAC ➣ **Enhancing trafficking and persistence within the immunosuppressive TME:** -­ CAR-CD147 construct -­ CCL19-expressing CAR-macrophages -­ Combination therapy with anti-CD47, anti-CD20 and anti-TAA antibodies

#### Overcoming tumor antigens heterogeneity, tumor antigens loss and scFv-based CAR-T cell limitations

An important challenge for CAR-T cells’ design and development is to find the appropriate antigen that is uniquely expressed by tumor cells and not by benign tissues. CARs targeting more than one antigen are being tested to overcome antigen loss variants of sub-clonal populations. Pooled CAR-T cell strategies consist of using two or more different CAR-T cells together, each targeting a single antigen. This strategy targets tumor cells in case of antigen loss, decreasing the chances of tumors resistance. Indeed, the combination of EGFR- and CD133-specific CAR-T cells showed improved outcomes in cholangiocarcinoma [88]. Additionally, in a NSCLC model, the combination of prostate stem cell antigen (PSCA)- and MUC1-targeting CAR-T cells synergistically eliminated PSCA^+^ and MUC1^+^ cancer cells [89]. A similar approach was applied for lung cancer by pooling EphA2-targeting CAR-T cells against tumor cells and fibroblast activation protein-α (FAPα)-targeting CAR-T cells against FAP^+^ stromal cells. This strategy was meant to kill cancer cells and simultaneously decrease the immunosuppressive function of FAP^+^ stromal cells in the tumor microenvironment (TME) [90]. This combinatorial strategy demonstrated significant tumor killing in vitro and extended the survival of mouse xenografts compared to each CAR-T cell therapy alone [90].

Multiplexing CAR strategy can also include dual tumor antigens targeted by bispecific-CAR-T cells (biCAR-T). Interestingly, using a combination therapy targeting IL13Rα2 and HER2 by bispecific CAR-T cells co-expressing IL13Rα2 and HER2 CAR molecules demonstrated significant potential for eliminating solid tumor cells and showed less antigen escape compared with mono-specific or pooled HER2-CAR-T and IL13Rα2-CAR-T cells alone in a glioblastoma model [91]. In breast cancer, biCAR-T cells targeting ErbB2 and MUC1 in vitro, showed efficient antitumor activity [92]. The development of biCAR-T cells with dissociated signaling pathways connected to a costimulatory signal and an activation signal is another promising strategy to improve T cells’ specificity. In this case, T-cell activation signal is physically dissociated from the costimulatory signal in two different CARs. Hence, biCAR-T cells become activated only when they simultaneously encounter two specific tumor cell antigens by tumor cells [84]. Some studies have proposed approaches to switch on or off CAR-T cells. Such strategies provide an accurate control of CAR-T cells activation and inhibition if toxic reactions arise [93]. For instance, using a bifunctional small “switch” molecule, which is composed of folate and fluorescein isothiocyanate (folate-FITC), allowed CAR-T cells to specifically identify tumor cells overexpressing folate receptors [94]. In addition, using suicide genes or antibody-mediated killing would shut-down CAR-T cells activity. Indeed, incorporating the inducible caspase 9 (iCasp9) system into CAR-T cells induced apoptosis leading to a repression of CAR-T cells activity [95]. Interestingly, using CAR-T cells targeting glycosylated antigens that are expressed on cancer cells, is also an interesting approach to overcome the tumor-immune response escape [96]. The success of CAR-T cell cocktails described above confronts related toxicities to be further investigated for fully evaluating clinical safety, particularly regarding OTOT toxicity [86] that may cause damage to healthy cells and organs. The risk of OTOT is enhanced by using more specific multi-antigens targeting CAR-T cells [87]. Therefore, controlling “on-target/off-tumor” (OTOT) toxicity during CAR T-cell therapy is one of the most important current challenges for optimal success of this new treatment strategy.

Structurally, CAR molecule is composed of an ectodomain, transmembrane domain, one or two costimulatory domains, and an activation domain [97]. The ectodomain is the extracellular section of CAR molecule in which a targeting domain can recognize antigens [98]. Single-chain variable fragment (scFv) is the most common targeting domain of CARs. It is responsible of recognizing the cell surface target antigens of interest and it mediates specific cytotoxicity against cells expressing these antigens [99–101]. However, Multiple limitations that can appear as obstacles to the safety and efficacy of CAR-T products are related to their targeting domains such as scFvs [99, 102]. These limitations of scFv-based CAR-T cells including the emergence of anti-idiotypic responses against the CAR targeting domain, and scFv aggregation resulting in pre-mature and antigen-independent CAR-T exhaustion can be overcome using nanobody-based CAR-T cell therapy. In different in vitro preclinical xenograft models, and in clinical studies, VHH-based CAR-T cells exhibited target antigen-dependent cytotoxicity against various types of malignancies [102].

#### Facilitating CAR-T cells tumor infiltration

Before antigen recognition, CAR-T cells need to successfully access the tumor site. CAR-T cells migration depends on chemokines secreted by tumor cells and chemokine receptors (CCRs) expressed by effector T-cells. Therefore, optimizing CAR-T cell therapy expressing appropriate CCRs that are capable of binding to specific chemokines secreted by tumors would promote their infiltration into the tumor microenvironment. Along this line, it has been shown that T-cells engineered with the chemokine receptor CXCR2, binding to the ligand CXCL1 on melanoma cells, had an effective trafficking effect to the tumor site [103]. Accordingly, in malignant pleural mesothelioma and neuroblastoma, tumor infiltration was improved through CCR2b-expression in mesothelin- and GD2-targeting CAR-T cells, respectively [104]. Several studies demonstrated the involvement of IL-8 (CCL8) as a pro-inflammatory chemokine promoting angiogenesis and tumorigenesis in many cancer types including prostate [105], ovarian [106], breast [107] melanoma [108] and colon [109]. Thus, researchers have generated a CAR-T cell strategy capable of expressing IL-8 receptors (CXCR1 or CXCR2) thereby enhancing their capacity of infiltrating solid tumors, consequently exerting an anti-tumor effect. Data of this research conducted on solid tumors in mouse models showed increased CAR-T cell tumor infiltration and persistence, with significant tumor toxicity [110, 111]. In a preclinical study, CX3CR1-expressing CAR-T cells showed significantly enhanced trafficking of CX3CL1-producing tumor cells accompanied with cancer cell regression [112]. In another preclinical study, Lo et al. have engineered CAR-T cells expressing macrophage colony-stimulating factor- 1 receptor (CSF-1R) binding to CSF1, a monocyte-recruiting chemokine synthetized by tumor cells, thereby enhancing CAR-T cells infiltration [113].

Another strategy which has recently been evaluated is the design of CD39- expressing CAR-T cells with triple shRNA knockdown of mucin domain-3 (TIM-3), lymphocyte-activation gene and PD-1, T-cell immunoglobulin domain-3 (LAG-3) to enhance their infiltration to the tumor site. CD39 is an extracellular ATP hydrolase enzyme expressed by CD8^+^ T cells; its expression is necessary for CAR-T cells cytotoxicity [114]. This evaluation showed that CD39 + -CAR-T cells had an enhanced antitumor effect in HCC organoids and PDX thereby improving migration to the tumor. Moreover, local application of CAR-T cells, such as intraperitoneal and intra-tumoral injection, would likely increase their accumulation at the tumor site. Local application via the peritoneal and pleural cavities has also been effective in ovarian cancer and malignant pleural mesothelioma [115]. Recently, Hongjun et al. have implemented a transdermal porous microneedle patch allowing the intra-tumoral penetration of CAR-T cells and enhancing their infiltration, as compared to direct intra-tumoral injection in solid tumor [116]. Moreover, local CAR-T cell administration prevented adverse effects associated with on-target, off-tumor responses, and lowered occurrence of Cytokine Release Syndrome (CRS) [117]. However, this approach is thus far limited by its high technical complexity and optimal delivery approaches necessary for patients with solid tumors that are unattainable to local delivery, such as brain and bone tumors [118].

Among the obstacles that circumvent CAR-T cells infiltration to the tumor site is abnormal vascularization which serves as an oxygen and nutrition source for tumors and also as a principal support for the circulation of tumors to other organs [97, 119]. To overcome the poor vascularization, recent studies have been targeting the vascular stroma instead of directly targeting cancer cells using anti-angiogenic molecules which are present in many types of cancers. Examples include vascular endothelial growth factor receptor 2 (VEGFR-2) in metastatic melanoma and other solid tumors [120, 121], VEGFR-1 in lung cancer [122], αvβ3 integrin in metastatic melanoma [123], αvβ6 integrin in cholangiocarcinoma (CCA) [124], ovarian, breast and pancreatic cancer [125]. An example of this is VEGFR-2-specific CAR-T generated against VEGFR2^+^ cells in the tumor vasculature [121]. The trial was effective in improving CAR-T cells infiltration and decreasing the growth of several vascularized syngeneic tumors of various sources [121]. A study conducted by Wang et al. showed that VEGFR-1 CAR-T cells can inhibit the resistance to traditional therapies targeting angiogenesis and provide CAR-T cells with tumor-killing ability [122]. Notably, the expression of integrin αvβ3 on activated endothelial cells and neo-vessels, but not on normal tissues, makes it an ideal target against many solid tumors [126]. In preclinical models, Wallstabe et al. demonstrated inhibition of tumor growth using αvβ3^+^ CAR-T cells [123].. The study also showed that results were improved when αvβ3^+^ CAR-T cells were combined with anti-avb3 mAbs [123].

Another potential approach besides recognizing stromal cell-associated antigens, is to enhance migration and infiltration capacity of CAR cells via disrupting physical barriers in solid tumors by designing CAR-T cells secreting matrix-degrading enzymes. Studies have shown that targeting CAR-T cells to fibroblast activation protein (FAP) can remove stromal cells, and engineering CAR-T cells secreting Heparinase enzyme (HPSE) can degrade the tumor matrix thereby overcoming tissue barriers [127–129]. Interestingly, scientists have discovered and are exploring ways to overcome the obstacle that solid tumors shield themselves in a “sugar coat”; sugars (glycans) on the surface of cells that renders themselves resistant to CAR-T cell attack. This has led to designing a molecular “torpedo” that can break the sugar shield thereby clearing a path for CAR-T cells to invade and destroy solid cancers [130]. The improvement of redirecting CAR-T cells to tumor cells is also achieved by modifying the design of CARs, e.g., Hinge domain, transmembrane domain and co-stimulatory signaling [131, 132]. Alternative non-LV or RV transduction and in vivo delivery of CARs [133]. Another strategy to increase CAR-T cell infiltration and counteract the immunosuppressive tumor microenvironment (TME) is to combine CAR-T cell therapy with other therapies like immune-checkpoint blockade. This combinatorial concept is detailed in subchapter 4 of this review.

#### Overcoming the immunosuppressive tumor microenvironment

Another challenge facing CAR-T cells in solid tumors, is the immunosuppressive TME. Indeed, once they reach the tumor, CAR-T cells must overcome a complex microenvironment structure with altered extracellular matrices (ECM), variable interstitial fluid pressure, hypoxic regions [134], immunosuppressive cells, regulatory T cells (Tregs), myeloid-derived suppressor cells (MDSCs), and tumor-associated macrophages (TAMs) for example [97]. Therefore, targeting immunosuppressive cells in the TME may improve efficacy of CAR-T cell therapy. Overcoming the immunosuppressive TME by developing armored CAR-T cells secreting immunostimulatory cytokines such as IL-12, IL-18, or IL-15, for example, modulates an immunomodulatory microenvironment leading to better CAR-T cell survival and to the recruitment of endogenous immune cells such as stem cells-like T-memory cells and central-memory T cells better fit for in vivo proliferation, survival, and persistence and to the recruitment of NK cells [135, 136]. Indeed, introduction of IL-12-secreting CAR-T cells resulted in increased anti-tumor immune response, especially by reducing CAR-T cell sensitivity to Treg inhibition [137] and also by reducing Tregs levels in the TME [138]. Similarly, IL-18- secreting CAR-T cells induced efficient antitumor immune responses by increasing the level of NK cells and M1 proinflammatory macrophages and by reducing CD103+ suppressive dendritic cells (DCs), and M2 anti-inflammatory macrophages density in the TME [139, 140].

Tregs are a main orchestrator of immune suppression in the TME through production of TGF-β (transforming growth factor-beta), which dampens the efficiency of immune effectors [141]. Therefore, different strategies have been conceived in deleting or inhibiting TGF-β receptor on the surface of CAR-T cells. Among others, CAR-T TGF-β dominant negative receptors (DNRs). In addition, swing receptors with chimeric signaling domains can convert TGF-β signals through engagement of the receptor modified to signal through co-stimulatory domains such as 4-1BB- or IL-12 stimulatory signals. Similarly, cytokine receptors containing the extracellular domain of the IL-4 receptor fused with the endo-domain of the IL-7 receptor turn swing suppressive into activating messages [142, 143]. Another strategy used recently is CRISPR gene editing technology to precisely insert the CAR in the genome of T cells [144]. To enhance CAR-T cells efficacy, CRISPER/Cas9 approach was used to knock out the endogenous TGF-β receptor-II (TGFBR2) gene in CAR-T cells, consequently inhibiting the effect of TGF-ꞵ and thereby reducing Treg cell induction and preventing CAR T cell depletion [145]. CRISPR can also be used to knock out the expression of PD-1 on the surface of CAR-T cells which can enhance their tumor-killing activity against PD-L1-expressing cancer cells, and prevent cancer relapse [146].

MDSCs can suppress the immune T cell response by various mechanisms such as impediment of disintegrin and the metalloproteinase- 17 (ADAM17) responsible for L-selectin-ectodomain cleavage, release of Nitric oxide (NO) and reactive oxygen species (ROS) [147, 148]. Hence, several strategies have been suggested to overcome the suppression of CAR-T cells by MDSCs. One of them is by decreasing the effects of ROS with all-trans retinoic acid (ATRA) [149]. Several combination therapies which address this obstacle will be described in subchapter 4.

Despite the important role of macrophages in the immune response against foreign pathogens, macrophages polarized towards an M2 phenotype play an anti-inflammatory and pro-tumor cell in the TME. Macrophages facilitate tumor progression and metastasis by promoting tumor cell invasion, angiogenesis, and immunosuppression [150, 151]. Several preclinical studies have targeted M2 macrophages with engineered CAR-T cells which can specifically deplete them. For example, folate receptor beta (FRβ)-specific CAR-T cells cause depletion of FRβ positive M2 cells in colon adenocarcinoma and melanoma [152]. A recent study conducted by Sanchez-Paulete and colleagues on a mouse orthotopic lung tumor model, showed that targeting the macrophage marker (F4/80) with F4-CAR-T cells delayed solid tumor progression, thereby enhancing anti-tumor immunity comparably to PD-1 blockade and prolonged animal survival [153]. The antitumor effect was also demonstrated in mouse models of pancreatic and ovarian cancer [153]. Additionally, repolarizing M2 into the proinflammatory M1 phenotype is a good strategy in order to reduce M2 macrophages and increase the antitumor M1 phenotype in the TME [154, 155].

#### Overcoming CAR-T cells’ toxicities

The cytokine release syndrome (CRS) is a common life-threatening inflammatory syndrome generated by overactivation of the immune response associated with CAR-T cell therapy. Cytokines, including interferon-γ (IFN-γ), IL-1, IL-6 and -10 have been associated with CAR-T cells-related CRS [156, 157]. To prevent CRS, many strategies have been developed including the administration of Anakinra, an IL-1 receptor (IL-1R) antagonist that demonstrated some effectiveness in treating CRS [158]. CAR-T cells secreting IL-1R antagonist have been constructed and suggested prevention of CRS-related mortality [159]. Also, IL-6 blockade by blocking IL-6R signals can decrease iNOS positive macrophage number and prevent CRS [160]. In addition, neutralizing granulocyte-macrophage colony-stimulating factor (GM-CSF), an important monocyte activator, could be an alternative approach for managing CRS as well as neurotoxicity [161]. An innovative study has shown that the release of cytokines and catecholamines, resulting from the interaction of CAR-T cells with the tumor, can be inhibited by catecholamine blockade with Atrial Natriuretic Peptide (ANP) [162].

Multiple other types of toxicity can occur in association with CAR-T cell treatment of solid tumors as well as hematological malignancies including on-target/off-tumor toxicity, neurological toxicity, anaphylaxis and graft versus host disease are also managed with different innovative strategies [163, 164]. Furthermore, the improvement of CARs, including controls of their activity and survival in vivo is considered as control of toxicities [165, 166].

In conclusion, the heterogeneous tumor antigen expression, lack of specific tumor antigen, limited tumor infiltration, and the immunosuppressive TME are the main challenges that impede the efficacy of CAR-T cell therapy in solid tumors. Further studies are required to improve CAR-T cell efficacy and toxicity by extending their persistence, facilitating their trafficking, and improving their infiltration to the tumor site. Furthermore, combination therapy with chemotherapy, radiotherapy and/or with other immunotherapies may improve CAR-T cell therapy in the future.

## CAR-NK cell therapy in solid tumors: applications, challenges and recent advances

Taking into consideration the shortcomings of CAR-T cells, there is a need to investigate other immune cells for CAR therapy, with increased attention on NK cells due to their immunological properties and their multiple sources [167]. Several advantages make NK cells an attractive alternative to CAR-T cells (Table 2).

### Advantages related to CAR-NK cell generation and manufacturing

For cancer immunotherapy, patient-derived NK cells function is usually hampered by curative treatments [168]. In fact, during tumor progression, the TME components can reduce NK cell capacity for proliferation, as well as ability of degranulation or cytokines secretion (such as TNF-α and IFN-γ) or expression of activating receptors [169]. Therefore, allogeneic NK cells are usually the first choice for cellular immunotherapy. Furthermore, while T cells are isolated from peripheral blood, either from the patient (autologous) or from a healthy donor (allogeneic), several sources have been used to generate allogeneic CAR-NK cells including peripheral blood (PB) from healthy donors, umbilical cord blood (UCB), induced pluripotent stem cells (iPSCs) or commercially available NK cell lines (NK92) [170]. Hence, “off-the-shelf” CAR-NK cells can be manufactured and infused to patients on-demand [171]. In addition, at least in theory, this type of production could reduce manufacturing costs and overcome the limited availability of autologous products in some malignancies, particularly in heavily pre-treated patients [172].

At least 90% of peripheral blood NK cells (PB-NK) are CD56^dim^CD16^bright^, representing a mature population with high cytotoxic potential [173, 174]. However, relatively few cells can be isolated from PB donors (around 10%) [175]. On the contrary, a high number of NK cells can be generated from umbilical cord blood (UCB) [176]. In addition, human-Embryonic Stem Cells (hESCs) and induced-Pluripotent Stem Cells (iPSCs)-derived NK can be generated in high quantity for immunotherapy use [177–179]. Consequently, the NK-92 cell line, isolated from a non-Hodgkin lymphoma patient, may be a potential source for limitless CAR-NK cells with high anti-tumor activity and direct cytotoxicity [180]. However, as NK92 cell lines are cytogenetically abnormal, they require irradiation prior to infusion with patients [181]. All these sources allow large-scale CAR-NK cell production to support multi-dose therapeutic infusion and on-demand cell product availability.

### Clinical application of CAR-NK cell therapy in solid tumors

NK cells possess advantageous characteristics, including non-MHC-restricted recognition, ability to infiltrate tumor tissues, cytolytic ability, minimal side effects (e.g., CRS, Graft versus host disease (GvHD) and Immune effector cell-associated neurotoxicity syndrome (ICANS). Therefore, CAR-NK cells can be considered an encouraging therapeutic option for the treatment of solid tumors. To date, only a few clinical studies evaluated NK92, PB-NK and UCB-NK based CAR-NK cell products with increasing interest in some commonly tumor targeted antigens such as Roundabout homolog 1 (ROBO1), NK cells activating receptor (NKG2D), MSLN, HER2 and MUC1 are registered in ClinicalTrials.gov and summarized in Table 3.

**Table 3:** Clinical trials of CAR-NK cell therapy in solid tumors (ClinicalTrials.gov)

CAR-NK Product	Clinical trial identifier	Targeted antigen	Disease	Cell source	Clinical trial phase	Status	Estimated enrollment (EE)/ Treated patients (TP)	Study objectives
ROBO1 CAR-NK cells	NCT03940820	ROBO1	Solid tumor	Human primary NK cells	Phase 1 Phase 2	Unknown	20	Evaluation of the safety and effectiveness ROBO1 CAR-NK cells to treat solid tumors.
MUC1 CAR-NK cells	NCT02839954	MUC1	MUC1 positive relapsed or refractory solid tumor	Human primary NK cells	Phase 1 Phase 2	Unknown	10 (EE) 8 (TP)	Evaluation of the safety and effectiveness of CAR-pNK cell immunotherapy in patients with MUC1 positive relapsed or refractory solid tumor.
BiCAR-NK cells ROBO1 CAR-NK cells	NCT03941457	ROBO1	Pancreatic cancer	NK92 cell line	Phase 1 Phase 2	Unknown	9 (EE)	Evaluation of the effect of ROBO1-specific BiCAR-NK cells on patients with pancreatic cancer.
Claudin6 CAR-NK cells	NCT05410717	Claudin6	Stage IV ovarian cancer Testis cancer Refractory endometrial cancer	Human primary NK cells	Phase 1 Phase 2	Recruiting	40 (EE)	Evaluation of the safety and preliminary efficacy of CLDN6-CAR-NK in patients with CLDN6-positive advanced solid tumors.
NKG2D-CAR-NK92 cells	NCT05528341	NKG2D	Relapsed/refractory solid tumors	NK92 cell line	Phase 1	Recruiting	20 (EE)	Clinical investigation of NKG2D-CAR-NK92 cells in the treatment of relapsed/refractory solid tumors.
NKG2DL CAR-NK cells	NCT03415100	NKG2D	Metastatic solid tumors	Autologous or allogeneic NK cells	Phase 1	Unknown	30 (EE) 3 (TP)	Study of NKG2D-Ligand CAR-NK cells in patients with metastatic solid tumors.
5 T4 CAR-NK	NCT05194709		Advanced solid tumors	N/A	Early Phase 1	Recruiting	40 (EE)	Study of Anti-5 T4 oncofetal trophoblast glycoprotein (5 T4) conjugated antibody redirecting CAR-NK cells in advanced solid tumors.
5 T4 CAR-NK Cells	NCT05137275	5 T4	Locally advanced or metastatic solid tumors	N/A	Early Phase 1	Recruiting	56 (EE)	Evaluation of the safety, tolerability, and efficacy of Anti-5 T4 CAR-raNK cell therapy in locally advanced or metastatic solid tumors.
Mesothelin CAR NK Cells	NCT03692637	Mesothelin	Epithelial ovarian cancer	Human primary NK cells	Early Phase 1	Unknown	30 (EE)	Investigation of the safety and efficacy of anti-Mesothelin CAR-NK cells with epithelial ovarian cancer.
PSMA CAR NK Cell	NCT03692663	PSMA	Metastatic castration-resistant prostate cancer	Human primary NK cells	Early Phase 1	Recruiting	9 (EE)	Evaluation of the safety, tolerability, and preliminary efficacy of TABP EIC in patients with metastatic castration-resistant prostate cancer.
HER2-CAR-NK	NCT03383978	HER2	Glioblastoma	NK92 cell line	Phase 1	Recruiting	42 (EE)	Study of intracranial injection of NK-92/5.28.z cells in combination with intravenous ezabenlimab in patients with recurrent HER2-positive glioblastoma.
CCCR-NK92	NCT03656705		Non-small cell lung carcinoma	NK-92 cell line	Phase 1	Enrolling by invitation	5 (EE)	Evaluation of the safety and efficacy of CCCR-modified NK92 infusions in previously treated advanced non-small cell lung carcinoma (NSCLC).
MUC1 CAR-pNK cells	NCT02839954	MUC1	Hepatocellular carcinoma Non-small cell lung cancer Pancreatic carcinoma Triple-negative invasive breast carcinoma Malignant glioma of the brain Colorectal carcinoma Gastric carcinoma	NK92 cell line	Phase 1 Phase 2	Unknown	10 (EE)	Evaluate the safety and efficacy of CAR-pNK cell immunotherapy in patients with MUC1 positive relapsed or refractory solid tumor.
MICA/B CAR-NK cells	NCT05395052	MICA/B	Non-Small cell lung cancer Colorectal cancer Breast cancer Ovarian cancer Pancreatic cancer Head and neck cancer Gastroesophageal cancer	Allogeneic natural killer	Phase 1	Recruiting	322 (EE)	Study of FT536 as monotherapy and in combination with monoclonal antibodies in subjects with advanced solid tumors.

Human primary NK cells have been tested in numerous clinical trials producing CAR-NK against specific tumor antigens, including ROBO1 for targeting several solid tumors (NCT03940820), PSMA for prostate cancer (NCT03692663), MSLN for epithelial ovarian cancer (NCT03692637) and Claudin6-for ovarian, testis cancer and refractory endometrial cancer (NCT05410717). Various clinical trials investigating CAR-NK92 cell therapy: HER2 CAR-NK targeted Glioblastoma (NCT03383978), and chimeric costimulatory converting receptor (CCCR) CAR-NK against non-small cell lung cancer (NCT03656705). MUC-1 specific CAR-NK cells are conceived for multiple relapsed or refractory solid tumors treatment (NCT02839954). In this study, of the 8 evaluable patients, seven achieved stable disease without serious adverse events [7]. Another study was performed to evaluate the clinical potential of a combination therapy using anti-ROBO1-specific biCAR-NK-92 in a patient with pancreatic cancer (NCT03941457). Another phase-I clinical trial (NCT05528341) investigated the effect of NKG2D CAR-NK92 cells for the treatment of relapsed/refractory solid tumors. Currently, a phase-I clinical trial (NCT03415100) was conducted recruiting patients with metastatic solid tumors to evaluate the safety of allogeneic or autologous NKG2DL-targeting CAR-NK cells transfected by mRNA electroporation [182]. Two early phase-I clinical trials (NCT05137275 and NCT05194709) targeting the 5 T4 oncofetal antigen in locally advanced or metastatic solid tumors by CAR-NK cells are currently recruiting patients.

On January 2022 the FDA allowed an investigational application for FT536 (by Fate Therapeutics) (NCT05395052), a CAR-NK cell therapy designed to treat patients with advanced solid tumors. FT536 is an allogeneic, multiplexed-engineered induced pluripotent stem cell-derived NK cell therapy genetically modified to targets the alpha-3 domain of the MHC class-I-related proteins-A (MICA) and -B (MICB). In addition, Benjamin H. et al. have recently described iPSC-NK cells as a promising alternative to T- cells for cellular therapy [183]. The promising conclusion was based on their proven safety profile, ability to be used as an allogeneic treatment and to be produced in large numbers to make an “off-the-shelf” therapy for the treatment of solid malignancies [183].

### Advantages related to mechanisms of CAR-NK cell recognition and killing of cancer cells

**Fig. 1:**
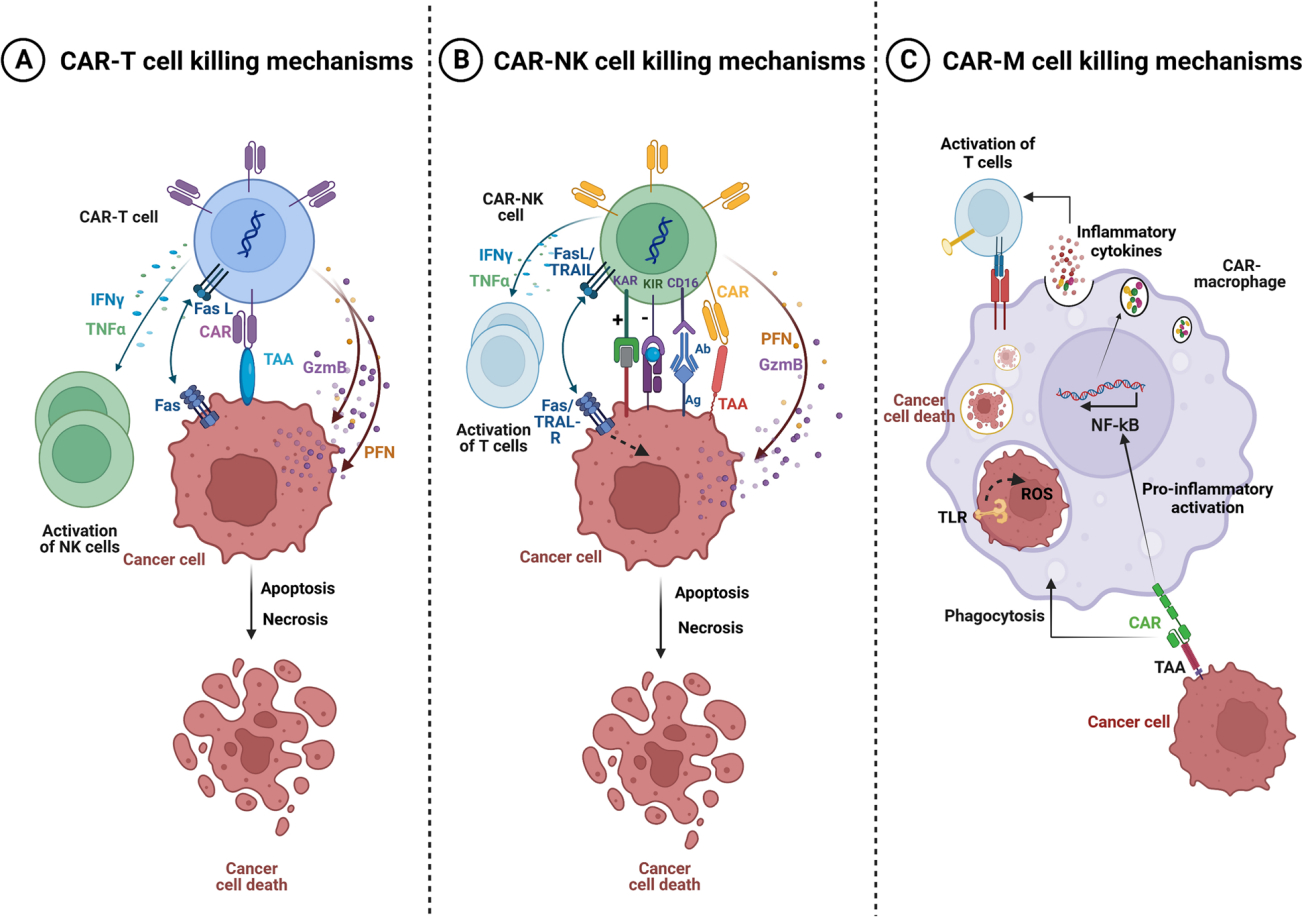
Killing mechanisms of CAR-T, CAR-NK, and CAR-M cells. **A** Tumor killing mechanisms of CAR-T cells. Activated CAR-T cells can specifically recognize the tumor associated antigen (TAA). Cytotoxic activity of Chimeric Antigen Receptor (CAR)-T cells is mediated by perforin (PFN) and granzyme (GzmB) granules secretion, and by activation of death receptor pathways such as Fas/Fas-L leading to cancer cells apoptosis and necrosis. Activated CAR-T cells also secrete Interferon-gamma (IFN-γ) and tumor necrosis factor-alpha (TNFα) which can promote Natural Killer (NK) cell anti-tumor cytotoxic activity. **B** Tumor killing mechanisms of CAR-NK cells. The activity of CAR-NK cells is regulated by the signal of activating (KAR) and inhibitory receptors (KIR) expressed on NK cells. Activated CAR-NK cells secrete the cytotoxic proteins perforin and granzyme B which synergize to induce cancer cell necrosis and apoptosis. NK cells also express the death ligands FasL and TRAIL which will bind to Fas and TRAIL-R on cancer cells and induce apoptosis. Moreover, CAR-NK cells trigger ADCC through the CD16 Fc receptor which recognize antibody-opsonized cancer cells. In addition, CAR-NK cells secrete IFN-γ and TNFα which promote their activation and stimulate other T-lymphocytes leading to increased anti-tumor immune response. NK: cell-Natural killer cells; IFN-γ: Interferon-gamma; TNFα: Tumor necrosis factor-alpha; TRAIL-R: TNF-related apoptosis-inducing ligand, KIR: Killer Inhibitory Receptors, KAR: Killer Activation Receptor, ADCC: Antibody-dependent cellular cytotoxicity, (PFN) perforin and (GzmB) granzyme. **C** Tumor killing mechanisms of CAR-M. The binding of a specific tumor associated antigen (TAA) with CAR receptor on the surface of CAR-M generates activation signals that mediate tumor phagocytosis, activation of transcription factors such as NF-kB and subsequent release of pro-inflammatory cytokines, which in turn can activate T cell-mediated immunity against the tumor

Human NK cells are innate cytotoxic immune cells that have been characterized by their “natural” ability to exert immune response to non-self-cells [184, 185]. NK cells use similar mechanisms to cytotoxic T-lymphocytes (CTLs) to kill cancer cells, but their target recognition mechanism is different [186, 187]. In fact, NK cells recognize malignant cells via multiple signals arising from different cell surface receptors, including activating and inhibitory killer cell Immunoglobulin-like receptors (KARs and KIRs) [188]. NK cells’ KIRs allows them to identify “self” [188], with this self-identification they can inhibit the cytotoxic activity against normal cells and prevent NK cell-derived “on-target, off-tumor” toxicity [189–191]. Moreover, while CAR-T cells only kill cells that have specific target antigens [192], CAR-NK cells exhibit intrinsic cytolytic activity, thus they would kill even cancer cells that do not express the target antigen [186] (Fig. 1A and B). In fact, CAR-NK cells still exert NK natural cytotoxic activity against tumor cells by the release of granzyme and perforin, for example, and can be activated via CAR-independent mechanisms, like natural cytotoxicity receptors (NCRs); NKp46, NKp44, and NKp30, NKG2D, co-stimulatory receptor; DNAX accessory molecule (DNAM-1), and specific activating KIRs (KIR2DS1, KIR2DS4 and KIR2DL4) [193, 194] which induce caspase-mediated apoptosis of targeted cancer cells. Moreover, NK cells can eradicate tumor cells by CD16-mediated Antibody-Dependent Cell-mediated Cytotoxicity (ADCC) [195]. Thus, CAR-NK cells would be able to efficiently eliminate tumors via both CAR-dependent and NK cells receptor-dependent mechanisms (Fig. 1B).

CAR-NK cells can recognize and kill tumor cells that don’t express MHC molecules [196] while reducing the risk of life-threatening GvHD and enabling allogeneic CAR-NK cell transplantations. Interestingly, unlike CAR-T cells, CAR-NK cells do not seem to cause severe toxicities such as CRS and ICANS [31]. This is partly due to a differential cytokine secretion profile, for example, activated NK cells usually produce IFN-γ and GM-CSF [197] whereas CAR-T cells predominantly produce IL-1, IL-6, Tumor Necrosis Factor-α (TNF-α) or Monocyte Chemoattractant Protein-1 (MCP-1), all which are associated with CRS and severe neurotoxicity [198]. Consequently, CAR-NK cells could be safer for clinical applications, compared to CAR-T cell products and toxicity.

### Limitations associated with CAR-NK cells and strategies to increase their effectiveness

Similar to CAR-T cells, CAR-NK cells face various obstacles in solid tumors such as migration to tumor site, persistence into the immunosuppressive TME and transduction [199]. Preclinical research is currently ongoing to optimize CAR-NK product in order to enhance their efficacy (see Table 2).

#### Generation of NK cell-specific CAR constructs to improve cytotoxic anti-tumor effects

In order to increase CAR-NK cells anti-tumor efficacy, several studies proposed to enrich CARs with certain domains associated with NK cell signaling such as NK-specific 2B4 and DNAX-activation proteins-10 or − 12 (DAP-10 or DAP-12) as co-stimulatory domains. This specific-NK cells construct showed greater cytotoxic effect and increased IFN-γ secretion compared to CAR-T cells constructs [200]. Armored CAR-NK with NKG2D receptor and costimulatory receptor 2B4 showed an increased cytotoxic effect in a xenograft ovarian mouse model expressing MSLN [201]. The result of this study was that MSLN-specific NKG2D.2B4.CD3ζ.CAR-NK cells induced higher tumor eradication and greater survival rate compared to the MSLN-specific CAR-T cells [201]. Furthermore, PSCA-specific CAR-NK cells equipped with DAP12, exerted higher anti-tumor activity compared to CD3ζ- CAR-NK cells in PSCA-positive tumor xenografts in immunodeficient mice [202].

#### NK cells specific CAR construct to improve in vivo survival and persistence within the TME

Another important challenge in CAR-NK generation is to extend their persistence in the peripheral blood and tissue. To achieve this goal, NK cells CAR construct can be armored with stimulatory cytokine-transgenes secreting for example IL-21, IL-15, IL-7 and IL-2 which promote NK cell proliferation and survival [203]. In order to preserve NK cells in vivo expansion after infusion, several feeders have been used such as autologous PBMCs, EBV-transformed lymphoblastoid cell lines (LCLs), and several NK cell-sensitive cell lines including K562 or 721.22 1[204, 205]. To overcome the immunosuppressive TME, chimeric co-stimulatory converting receptor (CCCR)-NK cells inhibited lung cancer growth in xenograft mouse models by switching the immunosuppressive negative PD-1 signal to an activating one [206].

#### Genetically engineered NK cells to improve trafficking to the tumor site

Müller and colleagues have shown that NK cells genetically engineered with EGFRvIII-specific CAR and a chemokine receptor CXCR4 have increased ability to infiltrate into the tumor site which can improve immunotherapy of solid tumors [207]. Another study investigated the transfection of NK cells with chemokine receptor CXCR1-mRNA construct and a CAR-mRNA construct against tumor-associated NKG2D ligands. The CXCR1-engineered NK cells showed enhanced in vitro migration toward tumor supernatants and increased in vivo infiltration into human tumors in subcutaneous and intraperitoneal xenograft models [208]. One of the major factors in the regulation of lymphocyte chemotaxis is CXCR3 expressed on activated NK cells inducing their migration toward chemokine ligands; CXCL9, − 10 and − 11. Therefore, CXCR3 receptor construct added to NK activating signaling domains may enhance the chemotaxis of NK cells to chemokine-secreting tumors and consequently improve their migration to the tumor site [209]. The engineering of NK- with CD19-CAR plus CXCR4 demonstrated in a pre-clinical model, the implementation of the migration of NK cells to bone marrow [210]. These findings suggest that the tumor infiltration can be improved in CAR-NK cell therapy and better clinical outcome can be expected.

#### New viral transduction enhancers for treatment of NK-cell-mediated CAR therapy

NK cells products are considered “hard-to-engineer” in comparison with T cells. To ameliorate NK cells transduction efficiency, lentiviral transductions have been significantly improved by incorporating new transduction enhancers which would help viral entry such as polybrene, (a cationic polymer frequently used to mediate viral entry into cell membranes) [211]. Retronectin has been described as a truncated version of fibronectin, which can colocalize cell’s surface with the virus [212] and vectofusin-1, a short cationic peptide which can considerably enhance the NK-cells viral transduction [213]. All of them may help CAR-NK engineering using viral transduction.

#### Electroporation and transposons for non-viral transduction of NK-cell mediated CAR therapy

Electroporation is a non-viral method to genetically engineer NK cells which promises 80 to 90% efficiency for mRNA-based plasmids but low efficiency for DNA electroporation [182, 214]. To introduce CAR constructs into the genomes of NK cells and ensure prolonged transgene expression, DNA transposons are most commonly used. These systems are composed of sleeping beauty (SB) subsets and the PiggyBac (PB) [201]. For instance, the generation of transposon-engineered CAR-NK cells, was found to be effective to achieve stable expression without viral integration in addition to other advantages including capacity for large gene fragment transduction, increased biosafety, low immunogenicity and worthful cost-effectiveness [215].

## CAR-M cell therapy in solid tumors: applications, challenges and recent advances

Currently, continuous investigational studies are trying to identify the ideal CAR cell type for targeting solid tumors. Macrophages have recently emerged as competitive candidates for the treatment of solid tumors due to their phagocytosis functional properties, antigen presentation and natural infiltration into the tumor microenvironment [34, 35] (Table 2).

### Advantages related to various sources of human CAR-macrophages production

Similar to CAR-NK, CAR-M can be generated from different sources including peripheral blood, iPSCs and the human leukemia monocytic cell line THP-1 (Table 4). PBMC derived M1 macrophages are characterized by their important production of proinflammatory factors such as IL-8, IL-6 and TNF-α [216], and a prominent expression of inflammatory surface markers such as natriuretic peptide receptor (NPR), CD14 and CD68 [217].. iPSCs can be induced into CAR-expressing macrophages (CAR-iMacs) exerting innate immune functions, such as repolarization of M2 phenotype into pro-inflammatory M1 in an antigen-dependent way, secretion of immune-related cytokines, as well as phagocytosis and antitumor capacity [218]. In addition, THP-1 cells can generate M1 macrophage after being stimulated with lipopolysaccharide (LPS) and IFN-γ and are easy to culture and to differentiate into macrophages [219–222].

**Table 4:** Comparison of CAR T, NK, and macrophages in solid tumors treatment

Criteria	CAR-T	CAR-NK	CAR-M
**Cell sources**	-­ PB (autologous/ allogeneic) -­ Rarely from iPSC and UCB	-­ UCB ­- BM -­ hESCs -­ IPSCs -­ NK92 cell line ­- PB	­- UCB ­- BM ­- hESCs ­- IPSCs ­- HPSCs ­- THP1 cell line ­- PB
**Availability**	-­ Autologous -­ MHC-matched allogeneic CAR-T cells -­ Unlikely “Off-the-shelf” product	-­ Autologous -­ Off-the-shelf CAR-NK product	-­ Autologous -­ Off-the-shelf CAR-M product
**In vivo safety and persistence**	-­ Long-term persistence, -­ On-target/off-tumor toxicity -­ GVHD, CRS and Neurotoxicity	-­ Limited lifespan -­ Reduced on-target/off-tumor toxicity -­ GVHD, no CRS nor Neurotoxicity.	-­ Limited time in circulation -­ Less On-target/off-tumor toxicity -­ No GVHD -­ CRS and Neurotoxicity
**FDA-regulated CAR-cell products**	-­ P-MUC1C-ALLO1 CAR-T cells (NCT05239143) -­ GD2 CAR T cells (NCT04196413) -­ GD2 CAR T cells (NCT00085930) -­ ROR1 CAR-T cells (LYL797) (NCT05274451) -­ ROR1 CAR-T cells (NCT02706392) -­ HER2 CAR-T cells (NCT03740256) -­ B7-H3 CAR-T cells (NCT04897321) -­ B7-H3 CAR-T cells (NCT04483778) -­ PSCA CAR-T cells (BPX-601) (NCT02744287) -­ PSCA CAR-T cells (NCT02744287)	-­ MICA/B CAR-NK cells (FT536) (NCT05395052)	-­ HER2 CAR-Macrophage (CT-0508) (NCT04660929)

Taken together, unlike CAR-T cells, CAR-M can be generated using several reliable sources. As an additional advantage, CAR-M bear a low risk of GvHD. Therefore, CAR-M approach might be an attractive allogeneic cell immunotherapy for solid tumors (Table 4).

### Clinical applications of CAR-macrophages for the treatment of solid tumors

To date, a few clinical trials of CAR-M are conducted and registered on clinicaltrials.gov (Table 5) and only one clinical trial of CAR-M has received the FDA approval. The first Phase I clinical trial (NCT04660929) CT-0508, a drug candidate from CARISMA Therapeutics, engineered with chimeric adenoviral vector Ad5f35 to target HER2 in solid tumors. In this study conducted by Klichinsky et al., the use of adenoviral infection induced macrophages differentiation into a pro-inflammatory M1 phenotype [223]. A phase-I clinical trial (NCT05007379) using HER2 CAR-M, is designed against organoids from breast cancer patients at different clinical stages. Another phase I clinical trial (NCT04405778) targeted Glypican 3 (GPC3), a protein expressed by some solid tumors but not expressed by normal cells, making it an ideal target for solid tumors. In this study, TAK-102, a GPC3 CAR-M, was tested in GPC3 positive solid tumors patients. Additionally, TAK-103, a MSLN specific CAR-M was also clinically tested (NCT05164666) in patients with MSLN-expressing advanced or metastatic solid tumors.

**Table 5:** Clinical trials of CAR-Macrophages cell therapy in solid tumors (ClinicalTrials.gov)

CAR-M Product	Clinical trial identifier	Targeted antigen	Disease	Cell source	Clinical trial phase	Status	Estimated enrollment (EE)	Study objectives
HER2 CAR- macrophages	NCT04660929	HER2	HER2 overexpressing solid tumors	Autologous macrophages (CT-0508)	Phase 1	Recruiting	48	Study of anti-HER2 CAR- macrophages in subjects with HER2 overexpressing solid tumors.
HER2 CAR-macrophages	NCT05007379	HER2	Breast cancer	N/A	Phase 1	Recruiting	100	Collection of tumor samples to develop patients’ derived organoids to test the antitumor activity of newly developed CAR-macrophages.
Glypican 3 (GPC3) CAR-macrophages	NCT04405778	GPC3	Solid tumors	N/A	Phase 1	Recruiting	18	Study of TAK-102 in adult patients with GPC3-expressing previously treated solid tumors.
Mesothelin CAR-macrophages	NCT05164666	Mesothelin	Advanced or metastatic solid tumors	Autologous white blood cells	Phase 1	Recruiting	21	Study of TAK-103 in adult patients with mesothelin-expressing advanced or metastatic solid tumors.

### Advantages related to the biological properties of macrophages

Tumor-associated macrophages (TAMs) often undergo activation into M1 (classical-activated macrophages) or M2 (alternative-activated macrophages) phenotype [224]. In terms of solid tumor therapies, M1 macrophages are involved in killing tumor cells by phagocytosis, reactive oxygen and nitrogen species (ROS/iNOS) release following the activation of Toll-Like Receptors (TLRs) [225] (Fig. 1C). In addition, M1 macrophages can release the pro-inflammatory IL-12 which initiates NK cells killing activity and stimulates both Th1 and tumor-specific CD8^+^ cytotoxic T cells responses [216, 217]. Furthermore, M1 macrophages can act as tumor antigen presenting cells inducing adaptive antitumor immune response [226]. Therefore, they are considered as anti-tumor or “good” macrophages [227] while M2 are considered as pro-tumor or “bad” macrophages [228]. For this reason, converting M2 TAMs into M1 macrophages is a promising immunotherapeutic approach for solid tumors [114]. Interestingly, CAR-M possess unique advantages over CAR-T and CAR-NK cells with regards to two major obstacles observed in solid tumors: ability to migrate and infiltrate into the immunosuppressive TME. In fact, in contrast to T cells poor infiltration, macrophages represent the predominant population of immune cells in the TME (reaching 50%) of various types of cancer such as melanoma, renal, and colorectal cancer [229]. In contrary to lymphocyte-based therapies, macrophages are able to remodel the extracellular matrix (ECM) [230]. Moreover, macrophages are an important source of matrix metalloproteinases (MMP) which degrade almost all ECM [33]. Taken together, CAR-M uses unique macrophage properties, especially phagocytosis which gives them a particular value over CAR-T and CAR-NK cell therapies. Consequently, CAR-M would have a significant potential in driving anti-tumor immunity in solid tumors.

### Limitations associated with CAR-M cells and strategies to optimize their use in solid tumor therapy

Several limitations are related to CAR-M bioengineering, storage, expansion, persistence at the TME, and toxicity (see Table 2).

#### Strategies to overcome the limitations in CAR-M bioengineering

Recent advances in gene transfection into effector cells have promoted diverse viral and non-viral engineering methods to overcome this challenge. Indeed, it has been shown that modified lentiviral virions containing Vpx; an accessory protein can efficiently deliver transgenes to myeloid cells [231]. In fact, Vpx, can mediate degradation of SAM domain and HD domain-containing protein 1 (SAMHD1); a myeloid-specific HIV-1 restriction factor that inhibits lentiviral transduction [232]. A supplementary option for macrophage transduction is the use of the chimeric Adenovirus 5-fiber 35 vector (Ad5f35) which can mediate efficient gene transfer into human macrophages [233]. In various studies, Ad5f35 showed a robust transduction of primary human macrophages [223, 234]. In addition, Ad5f35 infected macrophages activate the inflammasome and participate in maintaining the M1 phenotype generated by proinflammatory priming signals [235]. Additionally, transposon systems, mRNA transfection and bacterial plasmid DNA, have also been used as non-viral strategies for macrophages bioengineering [236–238]. Moreover, using polymer nanocarriers (mannose-conjugated polyethyleneimine (MPEI)), Kang and colleagues, were able to transfer the genes encoding CAR and IFN-γ into macrophages to enhance their anti-tumor potential [226].

#### Strategies to enhance the antitumor activity of CAR-M

In response to external stimuli, macrophages differentiate into antitumor proinflammatory M1. This concept prompted the first-in-human clinical trial conducted by Klichinsky et al. who demonstrated that anti-HER2 CAR-M efficiently induced phagocytosis of the HER2^+^ ovarian SKOV3 tumor cells, pro-inflammatory cytokines secretion, macrophages polarization from M2 to M1 phenotype and were capable of cross-presenting the New York Esophageal Squamous cell carcinoma 1 (NY-ESO-1) antigen to T cells, following NY-ESO-1^+^ SKOV3 tumor cells phagocytosis [223]. Moreover, Zhang et al., showed that CAR (MSLN)-iMacs can switch to the inflammatory M1 subtype and promote phagocytosis and immune activation when incubated in-vitro with MSLN-expressing ovarian (OVCAR3) and pancreatic (ASPC1), cancer cells [218]. This study also reported that CAR transgene expression was up to 85% in CAR-iMac cells [218].

#### Strategies to enhance trafficking and persistence of CAR-M within the immunosuppressive TME

Zhang’s CAR macrophages (CAR-147) consisting of scFv conjugated to a hinge region and CD147 transmembrane and intracellular domain to target HER2^+^ tumor cells effectively activated the expression of matrix metalloproteinases (MMP) such as MMP9, MMP10 and MMP12 [239–241]. Interestingly, this special CAR-CD147 construct can destroy the tumor’s extracellular matrix without affecting the phagocytic activity and inflammatory cytokines and ROS production [239, 240]. In addition, it was found that this CAR construct reduced the tumor growth and increased the T cell infiltration [239, 240]. Moreover, Niu et al. designed CAR-M to express CCR7 nature ligand, chemokine (C-C motif) ligand 19 (CCL19), in an attempt to target CCR7-expressing immunosuppressive cells [242]. The use of this CAR construct induced the of CD3^+^ T cells into tumors, increased pro-inflammatory cytokines production, suppressed tumor growth, decreased metastasis, and prolonged survival [242].

#### Overcoming CAR-M toxicity

Other hurdles faced by the CAR-M therapeutic approach are the potential to induce CRS and off target toxicity. Indeed, macrophages represent the principal source of the cytokine storms which can lead to CRS [243]. Additionally, since macrophages are distributed throughout the body, particularly in the liver [244] the use of CAR-M can lead to off target toxicity and limit efficacy. Therefore, further investigations are needed to optimize CAR-M production and ensure their safety.

## Potential combination therapies to enhance CAR-cell functions

Many studies demonstrated that CAR-cell monotherapy has limited efficacy for treating solid tumors [245–247]. Therefore, innovative combinations have been tested to synergize CAR-cell therapy.

### Combination with chemotherapy

**Fig. 2:**
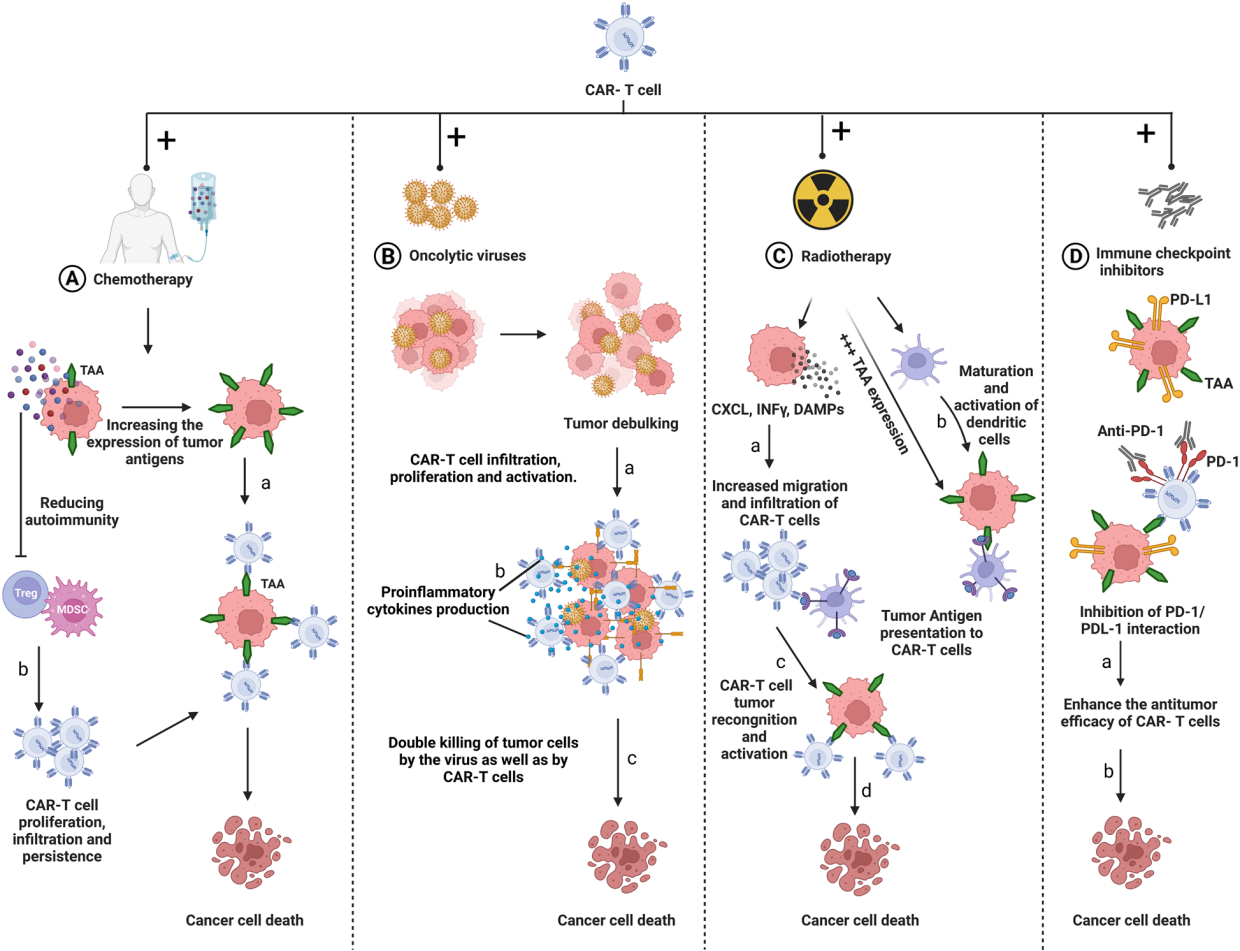
Possible combination therapies for CAR-T cells. **A** Combination of CAR-T cells with chemotherapy: Chemotherapy is known to increase the expression of tumor associated antigen (TAA) on cancer cells. This effect will help in (a) enhancing CAR-T cells interaction with cancer cells. Moreover, by downregulating regulatory T cells (Treg) and myeloid-derived suppressor cells (MDSCs), chemotherapy (b) promotes CAR-T cells proliferation, infiltration and extend their persistence in the TME. These mechanisms strongly support the use of CAR-T cells with chemotherapy for a more potent anti-tumor effect. **B** Combination of CAR-T cells with oncolytic viruses: Oncolytic viruses promote tumor debulking which (a) enhance CAR-T cells infiltration, proliferation, and activation (b) induce proinflammatory cytokines production, and (c) increase tumor cell death through a double mechanism: direct effect of the virus and enhanced CAR-T cells activity. **C** Combination of CAR-T cells with radiotherapy: Radiotherapy induces chemokines (CXCLs), interferon-gamma (INF-γ), damage-associated molecular patterns (DAMPs) release by tumor cells leading to (a) increased migration and infiltration of CAR-T cells. Radiotherapy also upregulates TAA expression on tumor cells allowing (b) maturation and activation of dendritic cells associated with better TAA presentation to T cells followed by (c) enhanced CAR-T cells tumor recognition and activation and leading to (d) increased cancer cells death. **D** Combination of CAR-T cells with immune checkpoint inhibitors (ICIs): ICIs targeting PD1/PDL-1 interaction unleash CAR-T cells inhibition by this repressive pathway. This effect will (a) enhance CAR-T cells cytotoxic activity and consequently (b) promote cancer cell death

When administered at low doses, chemotherapy plays an immunomodulatory role; it promotes dendritic cells activation and tumor antigen presentation to CAR-T cells, inhibits suppressive immune cells leading to increased persistence of CAR-T cells and sensitizes tumor cells to CAR-T cell activity by promoting granzyme B penetration into tumor cells [246, 248, 249] (Fig. 2A). Recently, Safarzadeh Kozani P et al. have reviewed in detail the positive effects of combining chemotherapy with CAR-T cell therapy [97]. This combination therapy can address the issue of CAR-T cells tumor trafficking to the TME resulting in more pronounced tumoricidal responses and increasing the rate of tumor rejection resulting higher survival rates [250]. Such positive effects have been attributed to the oxaliplatin-induced secretion of T cell-attractive chemokines by tumor associated macrophages resulting in improved CAR-T cell infiltration, remodeling of the tumor microenvironment, and increased tumor sensitivity to anti-PD-L1 [251].

**Fig. 3:**
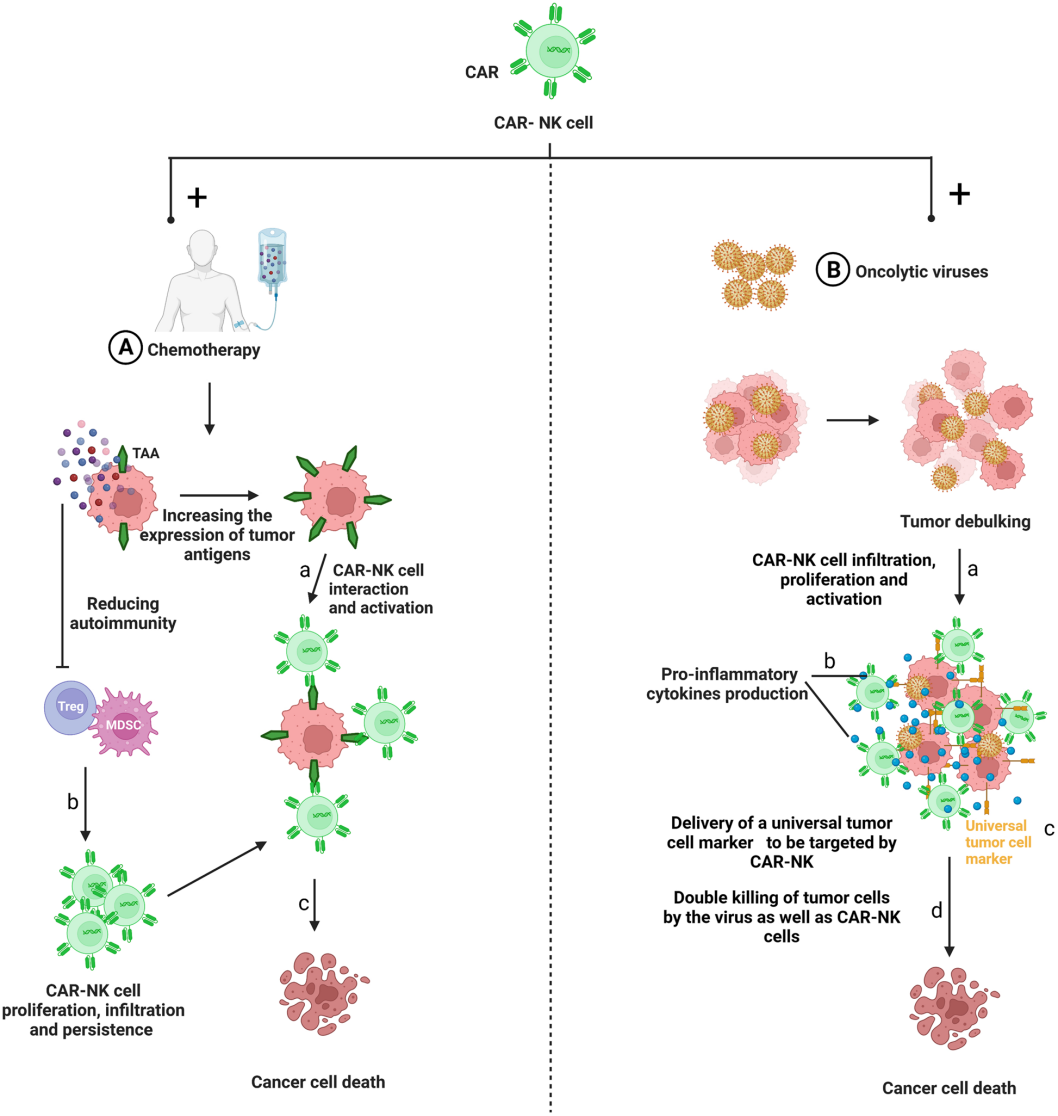
Possible combination therapies for CAR-NK cells. **A** Combination of CAR-NK cells with chemotherapy: The upregulation of tumor associated antigen (TAA) on cancer cells (a) enhances CAR-NK cells interaction with cancer cells and promotes their cytotoxic activity. Moreover, by decreasing Treg and MDSCs, chemotherapy (b) promotes CAR-NK cells proliferation, infiltration and prolongates their survival in the TME. Therefore, combination of CAR-NK cells with chemotherapy would (c) enhance tumor cell death. **B** Combination of CAR-NK cells with oncolytic viruses: The tumor debulking induced by oncolytic viruses (a) enhances CAR-NK cells infiltration, proliferation, and activation and (b) proinflammatory cytokines production. Chemotherapy can (c) deliver a universal tumor cell marker to be targeted by CAR-NK cells. Combination of CAR-NK cells with oncolytic viruses will (d) induce double killing of tumor cells by the virus and the CAR-NK cells

Importantly, it was demonstrated that sequential combination therapy with cisplatin followed by CD133-CAR-NK and CD44-CAR-NK92 cells led to the strongest killing effect on ovarian cancer stem cell lines compared to control NK cells [252, 253] (Fig. 3A).

### Combination with radiotherapy

Radiotherapy can directly kill cancer cells by apoptosis and necrosis, which induces dendritic cells maturation and activation and promotes tumor antigens’ presentation [254]. Following radiation, damage-associated molecular patterns (DAMPS) and INF-γ are released leading to an increased migration and infiltration of CAR-T cells into the tumor [255] (Fig. 2C). Combining CAR-T cell therapy with radiotherapy exerts a synergistic antitumor efficacy [97, 256].

### Combination with oncolytic viruses

Another combination strategy is the combination of CAR-T cells with an oncolytic virus [257, 258] (Fig. 2B). This combination overcomes major challenges that limits the efficacy of CAR-T cell therapy alone [259–261]. First, the virus can break through the tumor cells a difficult mission for CAR-T cell monotherapy. Second, the oncolytic virus can induce tumor debulking by destroying the molecular shield used by some solid tumors to escape the immune system attack. This effect may enhance CAR-T cells infiltration into the tumor site. Third, the oncolytic virus reverts the immunosuppressive TME to proinflammatory environment leading to increased proliferation and survival of CAR-T cells [259–261] (mechanisms illustrated in Fig. 3B). Several studies have demonstrated that this approach help increase the anti-tumor efficacy against solid tumors [262, 263].

### Combination with immune checkpoint inhibitors

It has been widely shown that PD-1 blocking antibodies, called immune checkpoint inhibitors (ICIs), would reinvigorate the CTL anti-tumor functions [264] (Fig. 2D). Combining CARs with ICIs demonstrated encouraging results. Interestingly, PD-1 blocking antibodies secreted by CAR-T cells themselves can competitively bind to PD-1 and enhances CAR-T cells proliferation and cytotoxicity [265–268].

**Fig. 4:**
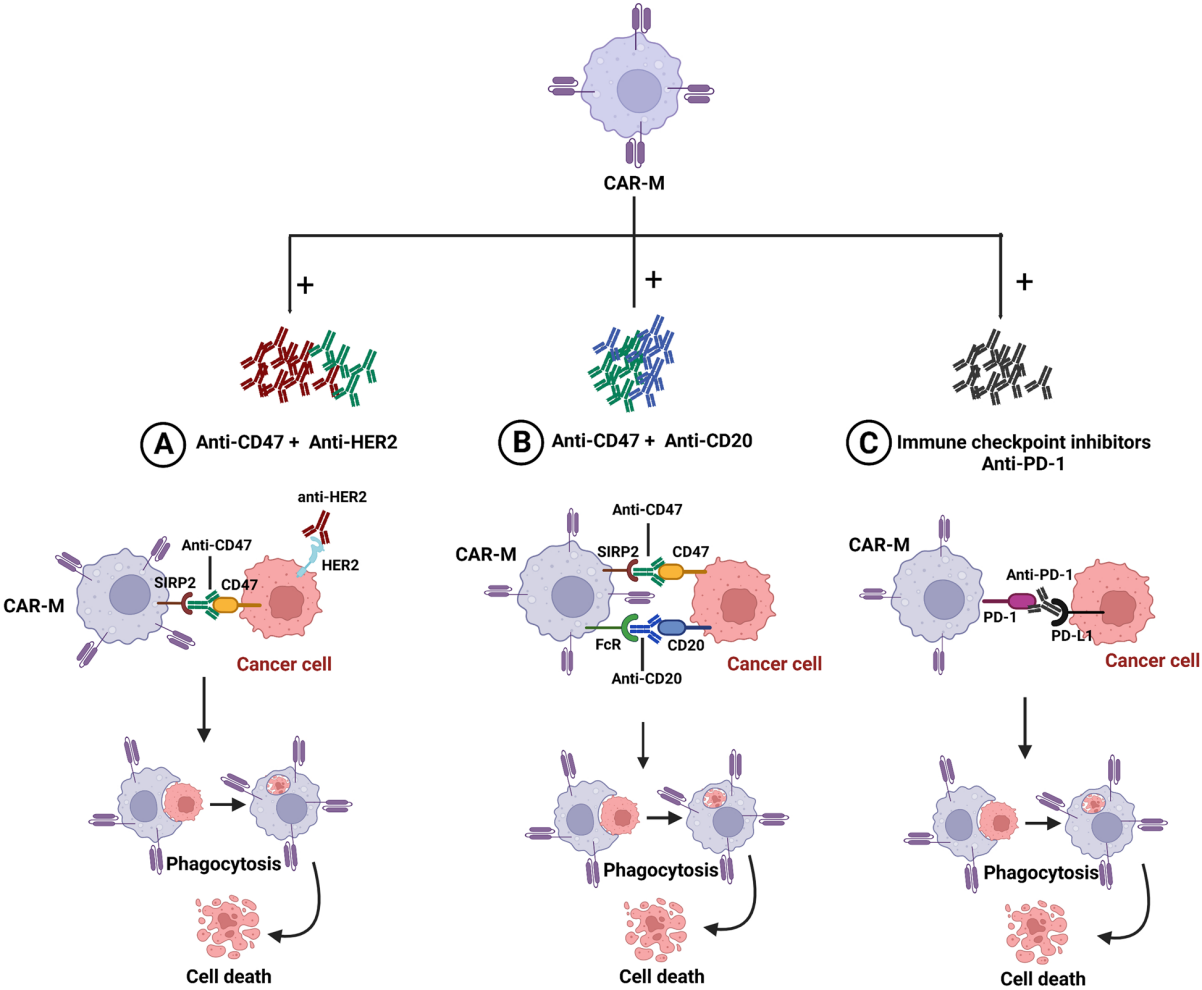
Possible combination therapies for CAR-M. CAR-M activation requires cancer cell recognition and interaction. Immune cell inhibitory mechanisms such as CD47/SIRPα or FcR/CD20 can limit CAR-M activity. CAR-M therapy demonstrated enhanced phagocytosis when combined with anti-CD47 and anti-HER2 (Trastuzumab) (**A**), with anti-CD47 and anti-CD20 (Rituximab) (**B**), as well as with anti-PD-1 (**C**). HER2; human epidermal growth factor receptor 2; PD-1; programmed cell death protein, SIRPα; signal regulatory protein α

A recent study showed that combining anti-PD-L1 monoclonal antibody with anti-PSMA CAR-NK-92 cells, enhances the antitumor efficacy against castration-resistant prostate cancer [269]. Furthermore, F Strassheimer et al. have demonstrated that combining CAR-NK cells with anti-PD-1 antibody enhances the cytotoxic T lymphocytes infiltration in the tumor site leading to a primed immune response and high tumoricidal activity against advanced-stage glioblastoma [270]. ICIs also improved macrophage phagocytic capabilities in vivo [271]. Combination therapy of CAR-M with PD-1 ICI, leads to synergistic tumor control and significantly increases overall survival in a syngeneic CT26 model [272]. In addition, antibodies blocking the interaction between CD47; overexpressed on many types of tumor cells and signal regulatory protein α (SIRPα) expressed in myeloid cells or the inhibitory Fc receptor FcγRIIB have been shown to enhance phagocytosis of macrophages [273] (Fig. 4).

### Combination with local tumor’ immunomodulating therapies

Local ablative therapies such as microwave (MWA) can destroy tumors causing hyperthermic damages in cancer cells and induces the release of immunomodulatory factors, such as danger signals, tumor antigens and cytokines responsible for stimulating an antitumor immune response [274]. A recent study demonstrated that combining MWA with CAR-T cells targeting a receptor tyrosine kinase (AXL) (AXL-CAR T cells) in NSCLC patient-derived xenografts, enhances the infiltration, activation, persistence, and tumor killing [275]. Photothermal ablation of the tumor combined with chondroitin sulfate proteoglycan-4 (CSPG4)-specific CAR T cells, demonstrated superior antitumor activity on melanoma WM115 cell line [276].

## Conclusion and perspectives

Recently, the clinical development of CAR-T cell therapy against solid tumors has tremendously evolved. However, some challenges facing CAR-T cell therapy in solid tumors are related to the tumor microenvironment such as: the lack of tumor-specific antigen, low efficiency of CAR-T cell trafficking, migration into tumor sites, and the presence of an immunosuppressive tumor microenvironment. Other major challenges are directly related to CAR-T cells including “on-target, off-tumor” toxicity, CRS, neurotoxicity and GvHD. These last limitations can be overcome using CAR-NK cell therapy which has been also preclinically well studied and translated to the clinical use. In fact, cytokines released by NK cells represent a diminished risk of CRS and neurotoxicity. Moreover, CAR-NK cells can be generated from different sources with reduced risk for alloreactivity and they can attack tumors through both CAR-dependent and CAR-independent manners which is considered as unique advantage of NK cells. However, some other challenges associated with CAR-T cells are also observed with CAR-NK cells including accessing the tumor tissue and resisting its immunosuppressive microenvironment. Advantages of CAR-NK therapy especially the reduced toxicities and their ability to produce a ready to use “off-the-shelf” product, make them a potential alternative to CAR-T cell therapy (Table 4).

**Fig. 5:**
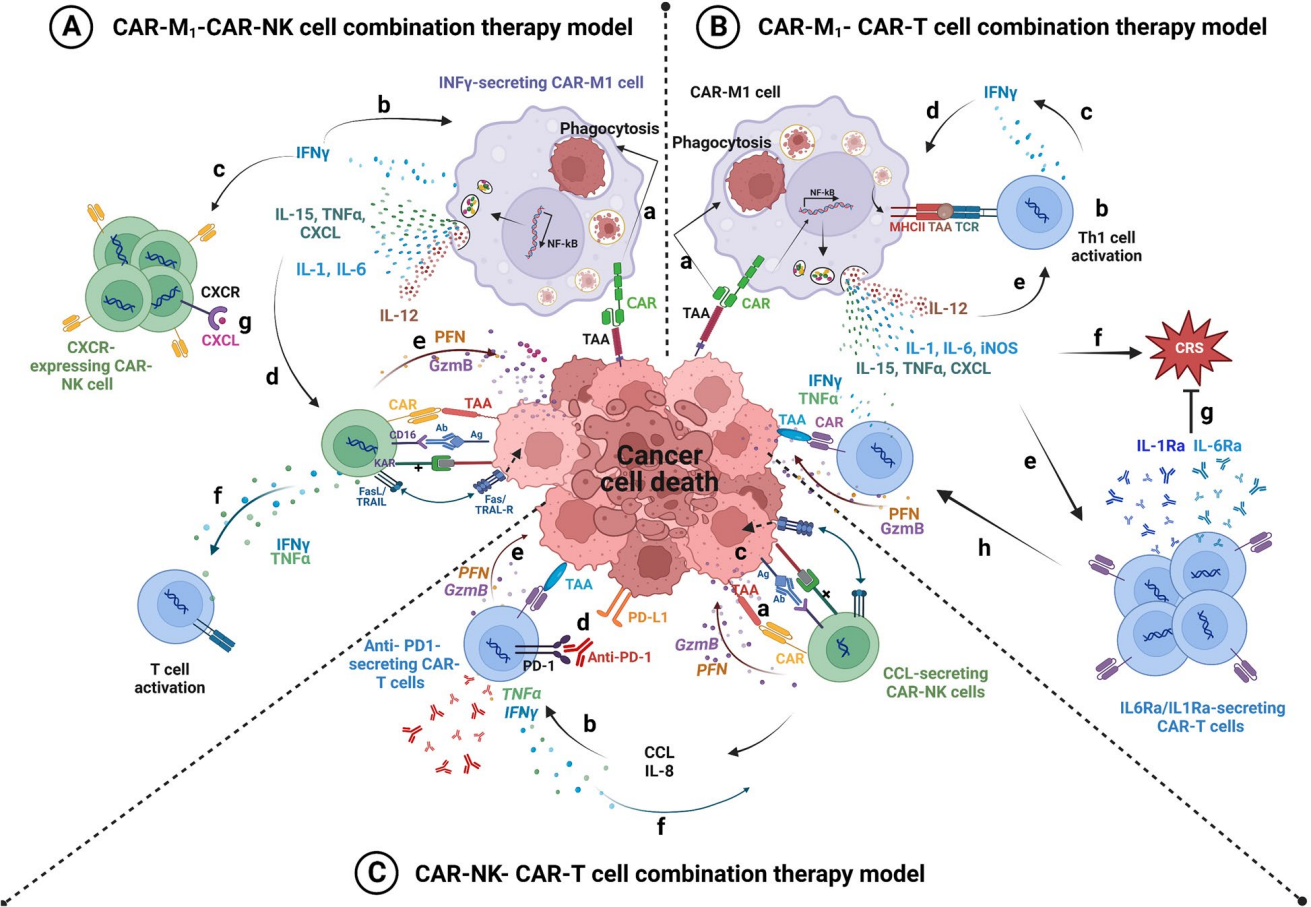
Combined CAR cells therapies would improve their efficacy. **A** CAR-M1/CAR-NK cell combination therapy model. (a) CAR-M1 genetically modified to secrete INF-γ recognize the TAA through the CAR and phagocyte the tumor. (b) The continuous secretion of INF-γ by these CAR-M maintains them in M1 phenotype and (c) induces the recruitment of CAR-NK cells genetically modified to express CXCR. Additionally, CAR-M1 secrete IL-1, IL-12 and IL-15 which (d) induce the upregulation of CAR-NK KAR), CXCR, FASL and CD16. The upregulation of activated CAR-NK cells (e) enhanced cytotoxicity against tumor cells. Additionally, activated CAR-NK cells secrete IFN-γ and TNFα which (f) stimulate endogenous cytotoxic T cells. Moreover, (g) CXCR-expressing CAR-NK cells have a higher potential to migrate and infiltrate the CXCL secreting TME. **B** CAR-M1/CAR-T cell combination therapy model. (a) CAR-M1 recognize TAA with CAR and phagocyte the tumor. TAA presentation by CAR-M1 induces (b) the activation of Th1 immune responses. The interaction between CAR-M1-MHC-TAA and Th1 induces (c) IFN-γ production by Th1. (d) IFN-γ maintains CAR-M in M1 phenotype. (e) Activated CAR-M1 cells produces pro-inflammatory cytokines and chemokines, ROS and TNF-α involved in the activation of Th1 and the recruitment of CAR-T cells into the tumor site. (f) CAR-M1 are also able to produce NO which contributes with IL-1 and IL-6 to the generation of CRS. (g) IL-1Ra-expressing-genetically modified CAR-T cells inhibit the CRC mediated by IL-1 and IL-6. (h) Recruited CAR-T cells recognize TAA and induce tumor cytotoxicity. **C** CAR-NK/CAR-T cell combination therapy model. (a) CAR-NK cells recognize TAA by CAR and ligands expressed on tumor cells. (b) CCL-secreting CAR-NK cells recruit CAR-T cells by releasing IL-8, CCL3, and CCL5. CAR-NK expressing chemokines are better recruited to cancer cells and (c) kill them directly by apoptosis. (d) CAR-T cells secrete PD-1 blocking antibodies and inhibits this interaction with PDL-1. (e) CAR-T cells induce cancer cells killing by releasing granzyme and perforin. (f) CAR-T cell recruit CAR-NK

As mentioned earlier in the previous parts of this review, blocking the PD1/PD-L1 axis by different strategies including PD1-blocking antibodies secreting CAR-T cells, lead to a better antitumor killing by CAR-T cells. In addition, CAR-NK cells secreting chemokines can recruit T cells. Considering these observations, we suggest combining PD1-blocking antibodies secreting CAR-T cells with CCL-CAR-NK cells could also be interesting to enhance CAR-T cell antigen recognition, interaction, and cytotoxicity against solid tumors (Fig. 5C).

CAR-M cell mediated therapy addresses key challenges faced by current CAR-T-cell therapy by engaging both the innate and adaptive immune systems thereby launching a multipronged attack against tumors. Recent findings have highlighted the importance in the interaction of modified or non-modified macrophages with T- or NK-cells in tumor regression [277]. Considering these advantages, we suggest combining CAR-M with CAR-NK cells or CAR-T cells to enhance their antitumor efficacy (Fig. 5A and B).

Importantly, the 3 models would provide the benefit of targeting different tumor antigens at the same time by each of these CAR-cell therapies. In addition, a particular advantage associated with the models in Fig. 5A and C is represented by the possibility of using allogeneic CAR-cells from different sources.

Researchers are currently looking to improve the efficacy of CAR-cell therapy by using various strategies including the Artificial Intelligence (AI) which could serve to counter many hurdles associated with CAR-cell therapy [278, 279]. In fact, radiomics, a quantitative approach to medical imaging may be useful for predicting novel cancer-associated antigens, new molecules in immune cells as well as analyzing safety and efficacy of CAR-cells [278, 279]. At the larger scale, AI can be used in automated CAR-T cell manufacturing which allows shorter production and delivery times to positively increase the number of patient treatments [280].

## Data Availability

Not applicable.

## Abbreviations

CAR-T cells: Chimeric antigen receptor T cells
CAR-NK cells: Chimeric antigen receptor natural killer cells
CAR-M cells: Chimeric antigen receptor macrophages cells
CD: Clusters of differentiation
CRS: Cytokine released syndrome
BCMA: B cell maturation antigen
HER2: Human epidermal growth factor receptor 2
BTCs: Biliary tract cancers
PCs: Pancreatic cancers
OS: Overall survival
CNS: Central nervous system
CCL2: C-C motif chemokine ligand 2
CXCL: C-X-C motif chemokine ligand
CXCR: C-X-C chemokine receptor
CCR: Chimeric costimulatory receptor
IL-13Rα2: Interleukin-13 receptor subunit Alpha2
GBM: Glioblastoma
GD2: Disialoganglioside
DIPG: Diffuse intrinsic pontine glioma
DMG: Diffuse midline glioma
LAG-3: Lymphocyte activation gene 3
PD-1: Programmed cell death protein 1
ROR1: Receptor tyrosine kinase like orphan receptor1
ORR: Overall response rate
PFS: Progression free survival
EGFR: Epidermal growth factor receptor
NSCLC: Non-small cell lung cancer
CEA: Carcinoembryonic antigen
CRC: Colorectal cancer
CRS: Cytokine release syndrome
PEDD: Pressure enabled drug delivery
MSLN: Mesothelin
OC: Ovarian cancer
IL: Interleukin
TSAs: Tumor specific antigens
TAAs: Tumor- associated antigens
PSCA: Prostate stem cell antigen
MUC1: Mucin 1
EphA2: Ephrin type-A receptor 2
FAP: Fibroblast activation protein
biCAR-T: Bispecific-CAR-T
iCasp9: Incorporating the inducible Caspase 9
OTOT: On-target/off-tumor
CX3CR1: CX3C motif chemokine receptor 1
CX3CL1: C-X3-C motif chemokine ligand 1
CSF-1R: Colony-stimulating factor 1 receptor
VEGFR: Vascular endothelial growth factor receptor
HPSE: Heparinase enzyme
TME: Tumor microenvironment
ECM: Extracellular matrices
MDSCs: Myeloid derived suppressor cells
TAMs: Tumor associated macrophages
TGF: Transforming growth factor
DNRs: Dominant negative receptors
NO: Nitric oxide
ROS: Reactive oxygen species
ATRA: All-trans retinoic acid
TLR: Toll like receptor
FRβ: Folate receptor beta
ANP: Atrial natriuretic peptide
CTLs: Cytotoxic T-lymphocytes
ICANS: Immune effector cell-associated neurotoxicity syndrome
TNF: Tumor necrosis factor
MCP: Monocyte chemoattractant protein
NCRs: Natural cytotoxicity receptors
ADCC: Antibody-dependent cell-mediated cytotoxicity
MHC: Major histocompatibility complex
GvHD: Graft versus host disease
APCs: Antigen presenting cells
PBMCs: Peripheral blood mononuclear cells
UCB: Umbilical cord blood
PB-NK: Peripheral blood NK cells
hESC: Human embryonic stem cells
iPSC: Induced pluripotent stem cells
ROBO1: Roundabout homolog 1
PSMA: Prostate specific membrane antigen
CCCR: co-stimulating conversion receptors
mRNA: Messenger RNA
FDA: Food and drug administration
mbIL-15: Membrane-bound interleukin − 15
LCLs: Lymphoblastoid cell lines
SDF-1α: Stromal cell-derived factor-1α
BaEV-LV: Baboon envelope pseudotyped lentivirus
CSF1: Colony stimulating factor 1
VEGF: Vascular endothelial growth factor
NPR: Natriuretic peptide receptor
LPS: Lipopolysaccharide
SAMHD1: SAM domain and HD domain-containing protein 1
HPSCs: Hematopoietic stem cells
NY-ESO-1: New York ESOophageal squamous cell carcinoma 1
MMP: Matrix metalloproteinases
DAMPS: Damage associated molecular patterns
EpCAM: Epithelial cell adhesion molecule
PD-L1: Programmed death-ligand 1
ICIs: Immune checkpoint inhibitors
CTL: Cytotoxic T lymphocytes
GEMs: Genetically engineered macrophages
